# The impact of community-based health insurance on the utilization of medically trained healthcare providers among informal workers in Bangladesh

**DOI:** 10.1371/journal.pone.0200265

**Published:** 2018-07-11

**Authors:** Sayem Ahmed, Abdur Razzaque Sarker, Marufa Sultana, Sanchita Chakrovorty, Mohammad Wahid Ahmed, Farzana Dorin, Andrew J. Mirelman, Ziaul Islam, Mohammad Hafizur Rahman, Louis W. Niessen, Clas Rehnberg, Jahangir A. M. Khan

**Affiliations:** 1 Health Economics and Financing Research Group, Universal health Coverage, Health Systems and Population Studies Division, icddr,b, Dhaka, Bangladesh; 2 Health Economics and Policy Research Group, Department of Learning, Informatics, Management and Ethics (LIME), Karolinska Institutet, Stockholm, Sweden; 3 Department of Management Science, University of Strathclyde, Glasgow, United Kingdom; 4 School of Health and Social Development, Deakin University, Melbourne, Australia; 5 Department of Agricultural Economics, Purdue University, West Lafayette, Indiana, United States of America; 6 Centre for Health Economics, University of York, York, United Kingdom; 7 Health Economics Unit, Ministry of Health and family Welfare, Dhaka, Bangladesh; 8 Department of Clinical Sciences, Liverpool School of Tropical Medicine, Liverpool, United Kingdom; Seoul National University College of Medicine, REPUBLIC OF KOREA

## Abstract

We aimed to estimate the impact of a Community-Based Health Insurance (CBHI) scheme on utilization of healthcare from medically trained providers (MTP) by informal workers. A quasi-experimental study was conducted where insured households were included in the intervention group and uninsured households in comparison group. In total 1,292 (646 insured and 646 uninsured) households were surveyed from Chandpur district comprising urban and rural areas after 1 year period of CBHI introduction. Matching of the characteristics of insured and uninsured groups was performed using a propensity score matching approach to minimize the observed baseline differences among the groups. Multilevel logistic regression model, with adjustment for individual and household characteristics was used for estimating association between healthcare utilization from the MTP and insurance enrolment. The utilization of healthcare from MTP was significantly higher in the insured group (50.7%) compared to the uninsured group (39.4%). The regression analysis demonstrated that the CBHI beneficiaries were 2.111 (95% CI: 1.458–3.079) times more likely to utilize healthcare from MTP.CBHI scheme increases the utilization of MTP among informal workers. Ensuring such healthcare for these workers and their dependents is a challenge in many low and middle income countries. The implementation and scale-up of CBHI schemes have the potential to address this challenge of universal health coverage.

## Introduction

Bangladesh has made significant advancement in essential public health services delivery which resulted in lower maternal and child mortality [1]. However, the government of Bangladesh spends little on health (7.13 USD per capita in 2014) by global standards [2]. Out-of-pocket (OOP) expenditures is 20.77 USD per capita in 2014 and it constitutes 67.0% of total healthcare expenditure[3]. Due to such payments 15.6% of households face catastrophic health expenditure and almost 5 million people fall into poverty every year [4,5].Further, among those who access healthcare, 41.6% utilize services from informal (e.g. village doctors, drug-sellers) and traditional providers (e.g. faith-based healers, Kabiraj) [6], which results in over-utilization of drugs and adverse effects of the treatment in many cases[7–9].

In order to achieve Universal Health Coverage(UHC), dependency on OOP payment should be reduced and for doing thisintroduction of prepayment healthcare financing mechanismism is important [10].The government of Bangladesh developed the first ever healthcare financing strategy for the country[11]. However, having a large proportion of informal workers in the labor force presents a major challenge for achieving UHC in Bangladesh as well as other low and middleincome countries (LMIC) [11–13]. The informal workers alone constitute 88% of the total labor force in Bangladesh and contribute to 64% of total GDP[14]. The Government of Bangladesh is currently piloting a tax funded scheme for those below the poverty line called *Shasthyo Shuroksha Karmasuchi*. Similar arrangement is difficult to implement for informal workers by the government or development partners, but critical, since this group constitutes largest portion of the population (56.2% of total population; 85.7 million) [11]. Covering this group of population through something akin to an equity fund will require a large amount of funds. Therefore, health insurance more specifically, CBHI scheme can generate additional healthcare resources for informal worker [2]. It is noticeable that 86.7% of informal workers were willing-to-pay on average 18.20 USD yearly for such kinds of health insurance schemes [15,16]. Considering the contribution of the informal workers to the economy of Bangladesh and their demand for health insurance schemes, an effort to attract these people towards self-financing through risk pooling mechanism for health is important. In order to address the healthcare and associated financing for informal workers, the government of Bangladesh recommended Community-Based Health Insurance (CBHI) schemesin the healthcare financing strategy [11]. For assuring access to healthcare of organized informal workers, a CBHI scheme was piloted in Chandpur sub-district area of Bangladesh by the research team in collaboration with an cooparative of informal worker.

### CBHI scheme

A CBHI scheme comprising agroup of informal workers was implemented through a cooperative, named “Labor Association for Social Protection”. The enrolment in the scheme was voluntary. The scheme did a number of marketing interventions (such as group meetings, and individual counselling by marketing staffs) to include members in the scheme. Under one membership for informal workers, the other members in his/her household were considered as beneficiaries. A brief description of the CBHI scheme under this study are presented below,

- Target population: Informal workers with low income and their household members in Chandpur sub-district (comprising urban and rural areas) of Bangladesh
- Implementation entity: Cooperative under the Ministry of Local Government and Rural Development
- Beneficiaries: Six members of each household entitled to health benefits for one membership card. The children under 5 were automatically enrolled in the scheme and not counted under the beneficiary limit.
- Benefit package: (Table 1)
- Premium:600 BDT (7.72 USD) per household per year which is 2.68% of the informal worker annual income 22,352 BDT (287.60 USD)[17]

**Table 1:** The service package of the CBHI scheme.

Services	Co-payment/description
***Health benefits***	
GP Consultation	30 BDT (Market price = 300 BDT^a^)
Medicine	20% discount from maximum retail price
Diagnostic tests	50% discount on market price
Specialist Doctor’s consultation	100 BDT (Market price = 500 BDT)
Hospitalization	Maximum 4,000 BDT per household per year
Periodic satellite clinics in remote rural areas	Free of charge
***Non-health benefits***	
Savings opportunity	▪ Minimum 10 BDT and maximum 100 BDT per week per household ▪ Member can withdraw saved amount with 10% interest after 1 year period
Training programs	▪ 3 months computer training for student member of the household with a cost 1,200 BDT (market price = 4,500BDT) ▪ 6 months sewing training for female workers (free of charge)

^a^1USD = 77.72 BDT

There was a uniform benefit package for all member of the CBHI scheme. The scheme provides health services to members through its own paramedic, doctors and contracted private healthcare facilities. A group of specialized doctors were contracted from private facilities. Per-case payment mechanism was employed for paying the specialized doctors and diagnostic center. The GPs under the scheme were paid through capitation approach. There were no other pooling fund schemes for healthcare in the community during the project period.

The policy question that arises is whether the CBHI scheme influences utilization of MTPs among informal workers. This article thus examines the impact of CBHI scheme on healthcare utilization from MTPs.

## Materials and methods

### Ethics statement

Informed written consent was taken from all interviewees, and confidentiality and anonymity were ensured. This study was approved by the Ethical Review Committee of the International Centre for Diarrhoeal Disease Research, Bangladesh (icddr,b).

### Study design

A quasi-experimental approach was employed to examine the effect of CBHI on healthcare utilization. Those who join the CBHI scheme are considered as ‘cases’. On the contrary, ‘comparisons’ are those who do not join the cooperative, but had similar observable characteristics through matching on: occupation (same occupation, for example, a rickshaw-puller or a farmer), household composition (presence of elderly persons aged 60 years and above, children under-five and female members of reproductive age), location (same village) and household income (10% deviation). Effort was given for best possible direct matching between case and comparison in terms of quantitative value of the matching variables. The enrolment process in this scheme was voluntary in nature. In this case, it is difficult to obtain a group of individuals as baseline group from a same time point for following-up to obtain before after difference. Therefore we employed a quasi-experimental approach with a case and a comparison group. This design was supported by propensity score matching analysis for reducing bias in baseline covariates.

### Study population and sample

This study was conducted in Chandpur Sadar Upazila. It consists of 9 Unions (areas under sub-district) and 7 of them are covered by the CBHI scheme. An earlier study observed that the healthcare utilization rate was 6.2% in the uninsured population,[18] and we are expecting 5% increment due to insurance [19]. Using this difference in healthcare utilization, 777 households from each of treatment and control groups were estimated considering the 90% power and 10% non-response rate [20,21]. In total 1,554 households were included in the sample. However 1,292 households (83.1% of total sample) responded to this survey which comprises 6,694 individuals (insured = 3,548, uninsured = 3,146). The household survey was conducted from April to June, 2014 after 1 year of CBHI scheme introduction.

### Data collection tool and variables

A structured questionnaire was administered in a face-to-face interview of household head of the insured and the uninsured households. The demographic characteristics of individual members and household socioeconomic characteristics were collected. For healthcare seeking of any household members in past 90 days, the type of healthcare provider that was utilized was obtained. Generally the informal workers sought healthcare from village doctors, drug-sellers, traditional healers, doctors, private clinics, medical colleges and district hospitals, sub-district health complexes and NGO clinics[22,23]. We considered all but first three providers as MTP since they employed medically well-educated staffs.

Household wealth status was categorized into five quintiles ordered from poorest to richest based on the asset variables (like, housing material, sanitation facilities, access to utility services, access to drinking water and assets). A principal component analysis (PCA) was conducted using these asset variables to estimate the asset score. This asset score was used for categorization of wealth status of the households[24]. Household size adjustment was done for estimating PCA score.

### Statistical approach

Healthcare utilization, measured as the number of visits/admissions in the past 90 days, was estimated and compared across ‘intervention’ and ‘comparison’ households. Descriptive statistics of healthcare utilization were presented stratified by several dimensions, such as income quintiles, occupations and geographic areas. A Chi-square test was done for testing any association of insurance status with the demographic characteristics and prevalence of illness in past 90 days. Independent sample t-test of proportion difference was carried out for testing if there was any significant difference in healthcare utilization from MTP between ‘intervention’ and ‘comparison’ groups.

In multilevel logistic regression model was used to predict the likelihood of healthcare utilization from MTP by health insurance status while controlling for demographic and household socioeconomic characteristics. We used this analysis to account for the hierarchical structure of the two levels of data[25]. The primary explanatory variable of interest in this analysis, membership in the CBHI scheme, was at the household level and the dependent variable healthcare utilization from MTP was at the individual level. As control variables, we included individual characteristics such as age, sex, education, illness frequency and type of illness and household characteristics such as wealth quintiles and household size. From this analysis, we estimated the significant difference in utilization of MTPs between intervention and comparison as well as magnitude of that difference. The model was specified as:

$${logit(Y}_{ij})=\beta X_{ij}+\gamma w_{j}+r_{ij}$$ (1)

Where, X_ij_ is a vector of characteristics of i^th^ participants living in j^th^ household and wj is a vector of household characteristics. The coefficient β characterize partial association between individual characteristics (like, age, gender, marital status, occupation, education, illness or symptoms suffered and inpatient care utilization) and utilization of healthcare from MTP whereas; *γ* characterizes the partial association between household characteristics (like, health insurance status, household size and wealth quintiles) and such healthcare utilization. The r_ij_ is an error term. We estimated the odds ratio and its 95% confidence interval from this analysis.

### Propensity score matching

**Fig 1:**
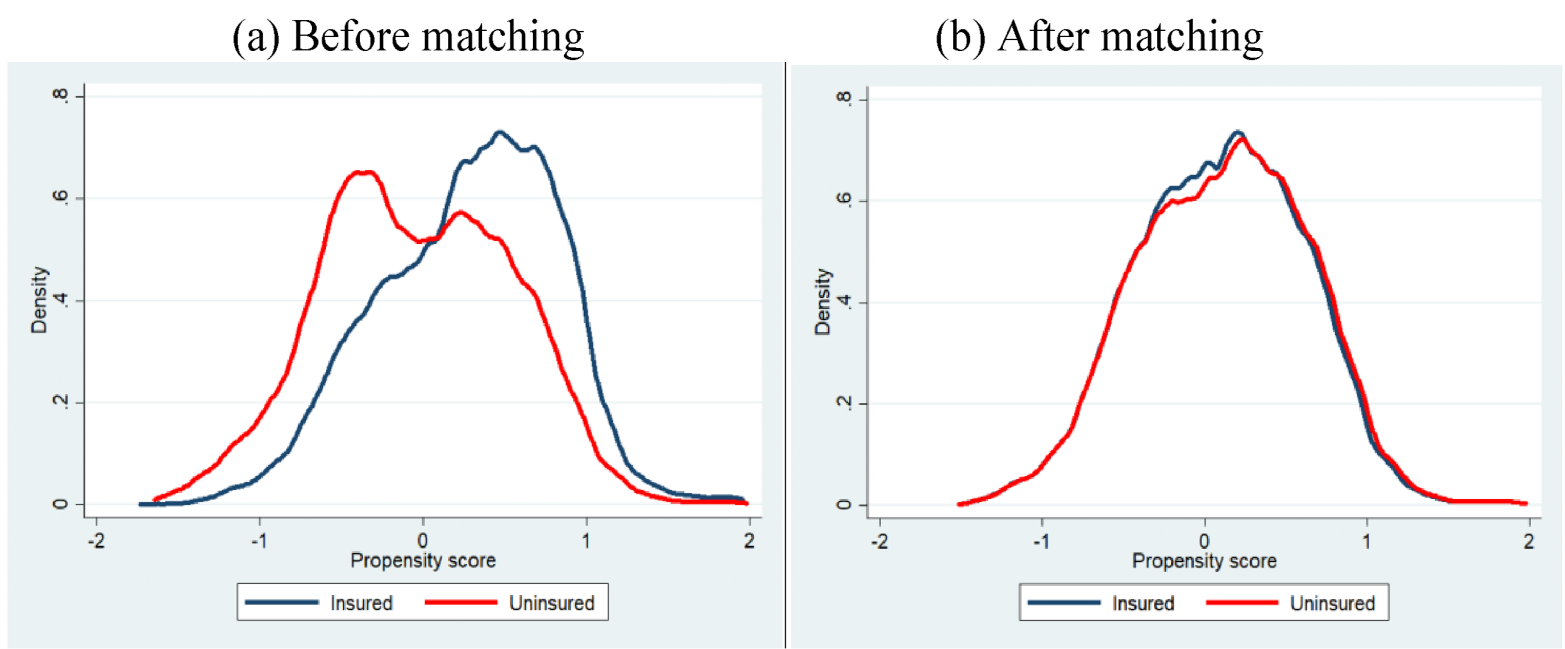
Propensity score distribution in the insured and uninsured groups before propensity score matching application and after matching.

Since we don’t have baseline information for intervention and comparison groups, baseline bias can exist after direct matching of household and individual characteristics. Therefore, to minimise the baseline difference in the characteristics a propensity score matching (PSM) approach was employed in estimating the impact of CBHI scheme on utilization of healthcare from MTP [26,27]. The PSM is a statistical tool which weight differences in observable variables between the individuals of insured and uninsured households. A logistic model was employed for estimating the propensity score. Based on the closeness of the estimated propensity score of each individual from insured group to the individual from uninsured group, a matched sample was drawn. The radius matching method was used to estimate the matched sample using recommended caliper size (standard deviation of the logit score is multiplied by 0.2)[28]. Fig 1 shows the propensity score distributions in the insured and the uninsured groups before propensity score matching application and after matching. Before propensity score adjustment the insured and uninsured group were dissimilar with regard to the characteristics measured by the propensity score, and after matching they are similar. After matching 2,519 individuals from each group were included in the analysis. In the matched sample, 639 household were from insured group and 611 households were from uninsured group.

The multilevel logistic model was applied on the matched observations to estimate the impact of CBHI on MTP provider utilization.

## Results

### Demographics and socioeconomic characteristics

A total of 3,548 insured (Male: 48.0%, Female: 52.0%) and 3,146 (Male: 46.4%, Female: 50.4%) uninsured household members were included in the study. The socio-demographic characteristics of the insured and uninsured participants are presented in Table 2. Before PSM matching, there were no significant differences between the age, gender, marital status and educational level of insured and uninsured participants at 5% significance level. However, there were significant associations of occupation, household size and asset quintiles with the insurance status without matching. In the post matched sample (2,519 individuals from each group) these association was found to be insignificant at 5% level of significance except for the age group between the insured and uninsured participants.

**Table 2:** Socio-demographic characteristics of the respondents.

Characteristics	Before matching			After matching		
	Insured	Uninsured	p-value^a^	Insured	Uninsured	p-value^a^
	% (95% CI)	%(95% CI)		%(95% CI)	%(95% CI)	
**Age group**						
Child (0–14)	30.0 (28.5–31.5)	32.3 (30.6–33.9)	0.091	29.6 (27.8–31.4)	32.9 (31.1–34.7)	0.039
Adult (15–60)	64.1 (62.5–65.6)	61.5 (59.8–63.2)		64.2 (62.3–66.0)	61.4 (59.5–63.3)	
Elderly(60+)	5.9 (5.2–6.7)	6.3 (5.4–7.1)		6.3 (5.4–7.3)	5.8 (4.9–6.7)	
**Sex**						
Male	48.0 (46.4–49.6)	49.6 (47.8–51.3)	0.204	50.4 (48.4–52.3)	48.2 (46.3–50.2)	0.128
Female	52.0 (50.4–53.6)	50.4 (48.7–52.2)		49.6 (47.7–51.6)	51.8 (49.8–53.7)	
**Marital status**						
Married	50.4 (48.7–52.0)	49.4 (47.6–51.1)	0.211	49.5 (47.6–51.5)	48.7 (46.8–50.7)	0.461
Unmarried	45.4 (43.8–47.0)	47.1 (45.3–48.8)		46.4 (44.5–48.4)	47.8 (45.8–49.7)	
Others (Widowed, Divorced and Separated)	4.2 (3.5–4.9)	3.6 (2.9–4.2)		4.0 (3.3–4.9)	3.5 (2.9–4.3)	
**Occupation**						
Agriculture worker	2.8 (2.2–3.3)	3.1 (2.5–3.7)	0.000	2.7 (2.2–3.5)	2.4 (1.9–3.1)	0.742
Labor	7.3 (6.4–8.1)	6.1 (5.2–6.9)		7.5 (6.6–8.6)	6.6 (5.6–7.6)	
Sales worker	4.4 (3.7–5.1)	6.3 (5.5–7.2)		5.2 (4.4–6.2)	5.5 (4.7–6.4)	
Service worker	5.5 (4.7–6.2)	7.0 (6.1–7.9)		6.6 (5.7–7.7)	6.1 (5.2–7.1)	
Housewife	23.4 (22.1–24.8)	23.0 (21.6–24.5)		22.9 (21.3–24.6)	23.2 (21.6–24.9)	
Transport worker	3.2 (2.6–3.7)	3.5 (2.9–4.2)		3.5 (2.9–4.3)	3.3 (2.7–4.1)	
Small business	2.0 (1.5–2.5)	2.2 (1.7–2.7)		2.1 (1.6–2.8)	2.2 (1.7–2.9)	
Not working/ unemployed	48.3 (46.7–50.0)	47.6 (45.8–49.3)		47.3 (45.4–49.3)	49.1 (47.2–51.1)	
Others	3.1 (2.6–3.7)	1.3 (0.9–1.7)		1.9 (1.5–2.6)	1.5 (1.1–2.1)	
**Household size**						
1–2 persons	3.4 (2.8–3.9)	9.0 (8.0–10.0)	0.00	4.7 (3.9–5.6)	4.3 (3.6–5.2)	0.649
3–4 persons	34.0 (32.4–35.5)	49.7 (47.9–51.4)		45.3 (43.3–47.2)	44.5 (42.5–46.4)	
5 persons or more	62.7 (61.1–64.2)	41.3 (39.6–43.0)		50.1 (48.1–52.0)	51.2 (49.3–53.2)	
**Education level**						
No institutional education	20.8 (19.4–22.1)	21.3 (19.8–22.7)	0.09	21.2 (19.6–22.8)	21.9 (20.3–23.5)	0.199
Primary level (years 1–5)	38.6 (37.0–40.2)	38.9 (37.2–40.6)		35.1 (33.3–37.0)	37.4 (35.6–39.3)	
Junior level (years 6–8)	23.6 (22.2–25.0)	22.3 (20.8–23.7)		25.9 (24.2–27.7)	22.9 (21.3–24.6)	
Secondary level (years 9–10)	11.3 (10.3–12.4)	12.0 (10.8–13.1)		12.5 (11.3–13.9)	12.4 (11.2–13.8)	
Higher Secondary level (years 11–12)	4.3 (3.7–5.0)	3.6 (2.9–4.2)		3.6 (2.9–4.4)	3.9 (3.2–4.7)	
Tertiary level (12+)	1.4 (1.0–1.7)	2.1 (1.6–2.6)		1.7 (1.2–2.2)	1.5 (1.1–2.0)	
**Location**						
Urban	33.9 (32.3–35.4)	33.0 (31.3–34.6)	0.43	35.1 (33.2–36.9)	34.0 (32.2–35.9)	0.441
Rural	66.1 (64.6–67.7)	67.0 (65.4–68.7)		64.9 (63.1–66.8)	66.0 (64.1–67.8)	
**Asset quintiles**						
Poorest	18.0 (16.7–19.3)	21.3 (19.9–22.8)	0.00	18.9 (17.4–20.4)	17.9 (16.5–19.5)	0.166
2^nd^	16.2 (15.0–17.4)	22.7 (21.3–24.2)		20.2 (18.7–21.9)	19.7 (18.2–21.3)	
3^rd^	19.6 (18.3–20.9)	19.7 (18.3–21.1)		19.6 (18.1–21.2)	20.9 (19.4–22.6)	
4^th^	24.0 (22.6–25.4)	16.9 (15.6–18.2)		17.9 (16.5–19.5)	19.9 (18.4–21.5)	
Richest	22.2 (20.8–23.6)	19.4 (18.0–20.8)		23.4 (21.8–25.1)	21.6 (20.0–23.2)	
**N**	**3,548**	**3,146**		**2,519**	**2,519**	**-**

^a^Chi-square test

### Utilization of healthcare

Table 3 represent the overall utilization of healthcare in last 90days period among the insured and uninsured groups. We found a significant difference (P = 0.013) in healthcare seeking behaviour of individuals who suffered illness. 97.7% of (815 individuals) insured individuals and 99.2% (786 individual) of uninsured individuals sought healthcare for their illness. A comparatively higher proportion of insured individuals (50.7%) than uninsured individual (39.4%) sought healthcare from MTP. In both insured and uninsured groups, the highest number of healthcare services were utilized from private providers (92.3% among insured and 90.7% among uninsured group) followed by public providers(5.9% in insured and 6.7% in uninsured group). The self-reported illness or symptoms in last 90 days were not-significantly associated with insurance status (P = 0.061). However, there was a mixed pattern of self-reported illness or symptoms between intervention and comparison individuals.

**Table 3:** Pattern of utilization of healthcare in the last three months.

Healthcare seeking/ illness	Insured	Uninsured		
	N	% (95% CI)	N	% (95% CI)	p-value
**Individual level sample (N)**	2,519		2,519		
**Suffered any illness or symptoms**					0.210^a^
No	1,685	66.9 (65.0–68.7)	1,727	68.6 (66.7–70.3)	
Yes	834	33.1 (31.3–35.0)	792	31.4 (29.7–33.3)	
**Seek healthcare among those who suffered illness**					0.0130^a^
No	19	2.3 (1.5–3.5)	6	0.8 (0.3–1.7)	
Yes	815	97.7 (96.5–98.5)	786	99.2 (98.3–99.7)	
**Seek healthcare from medically trained provider among those who sought healthcare**					0.0010^a^
No	402	49.3 (45.9–52.8)	476	60.6 (57.1–63.9)	
Yes	413	50.7 (47.2–54.1)	310	39.4 (36.1–42.9)	
**Self-reported illness/symptoms**					0.0610^b^
Communicable diseases	106	12.7 (10.6–15.2)	118	14.9 (12.6–17.6)	
Non-communicable diseases	122	14.6 (12.4–17.2)	117	14.8 (12.5–17.4)	
Accident and Injuries	21	2.5 (1.6–3.8)	28	3.5 (2.4–5.1)	
Female reproductive health problem and delivery care	25	3.0 (2.0–4.4)	14	1.8 (1.0–3.0)	
Symptoms	415	49.8 (46.4–53.2)	411	51.9 (48.4–55.4)	
Others	145	17.4 (15.0–20.1)	104	13.1 (10.9–15.7)	
**Healthcare provider utilized**					0.0790^b^
Public	48	5.9 (4.5–7.7)	53	6.7 (5.2–8.7)	
Private	752	92.3 (90.2–93.9)	713	90.7 (88.5–92.6)	
NGO	-	(-)	6	0.8 (0.3–1.7)	
Others (e g. traditional)	15	1.8 (1.1–3.0)	14	1.8 (1.1–3.0)	
**Inpatient care utilized**					0.260^a^
No	771	94.6 (92.8–96.0)	733	93.3 (91.3–94.8)	
Yes	44	5.4 (4.0–7.2)	53	6.7 (5.2–8.7)	

^a^ t-test.

^b^Chi-square test.

### Healthcare seeking behaviour

**Fig 2:**
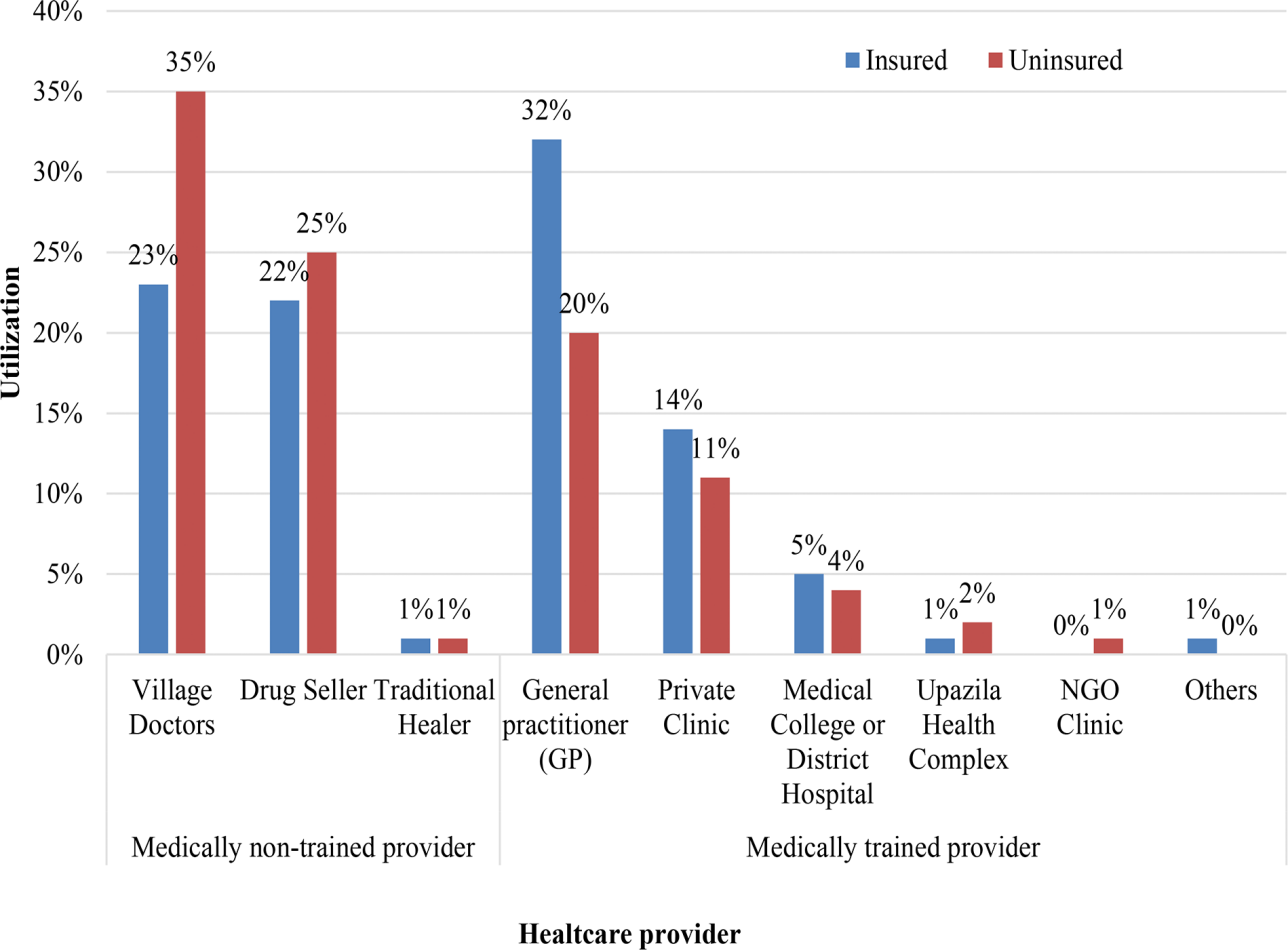
Healthcare seeking behaviour of CBHI scheme enrolees and uninsured group before matching.

Fig 2 presents the distribution of healthcare service utilization from different providers by insured and the uninsured. It was observed that the insured utilized village doctors and non-prescribed drug sellers by 12% and 3% less respectively than their corresponding uninsured. On the other hand, of total service utilization among CBHI scheme beneficiaries, 32% was to medically trained MBBS/specialist doctors, while 20% of such services were utilized by individuals in uninsured households. Utilizations of ‘private clinic’ and ‘Medical College hospitals and district hospitals’ were made by CBHI scheme beneficiaries at a higher proportion (14% and 5% respectively) than individuals in control households (11% and 4% respectively).

**Fig 3:**
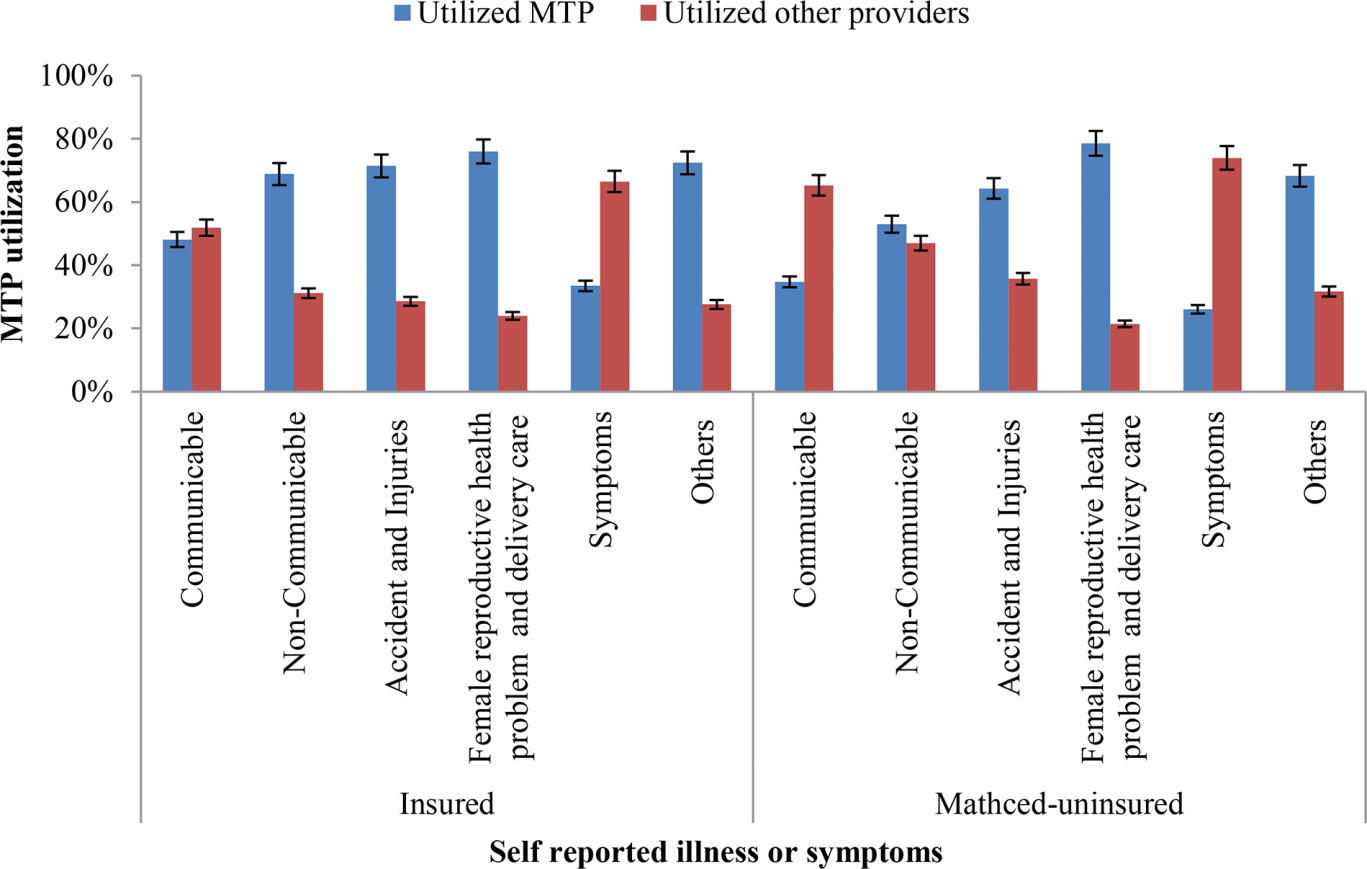
Medically trained providers utilization between insured and uninsured groups by self-reported illness or symptoms.

The utilization of MTPs and other providers between insured and uninsured groups by self-reported illness or symptoms are presented in Fig 3. The utilizations of MTPs were higher for non-communicable diseases, accident and injuries, female reproductive health problem and delivery care, other symptoms for both groups. However, for communicable disease the trained provider utilization were significantly lower in uninsured group. For different symptoms (e.g. fever, weakness) the trained provider utilization were lower in insured and uninsured groups.

### Econometric analysis

The multilevel logistic regression shows that the insured household members are 2.111 times more likely to utilize MTPs than the uninsured household members (Table 4). Such utilization was significantly less among unmarried household members (OR = 0.371; 95% CI: 0.186–0.774) than married members. Economic disparity was also observed in utilization of MTPs. Members of the richest householdwere6.954 times more likely to utilize healthcare than the members of poorest household. Inpatient healthcare services were more likely to be utilized (OR = 8.365; 95% CI: 3.659–19.13) from the MTPs. Individuals were more likely to utilize MTPs in the case of non-communicable diseases (OR = 2.823; 95% CI: 1.543–5.164), accident and injuries (OR = 3.969; 95% CI: 1.568–10.73), delivery care associated problems (OR = 6.204; 95% CI: 1.821–21.13), and in case of other illness (OR = 6.125; 95% CI: 3.236–11.60) rather than communicable diseases. However such utilization was less likely for symptoms (OR = 0.493; 95% CI: 0.305–0.796) than the communicable diseases.

**Table 4:** Estimated effect of CBHI scheme enrolment on utilization of medically trained healthcare providers.

		Dependent = Utilized medically trained provider
		OR (95% CI)
*Health insurance status*	Member (Ref = No membership)	2.111*** (1.448,3.079)
*Age-group*	Adult, 15–60 years (Ref = Child, 0–14 years)	0.907 (0.448,1.835)
	Elderly, 60+ (Ref = Child, 0–14 years)	0.301* (0.117,0.774)
*Gender*	Female (Ref = Male)	1.039 (0.657,1.644)
*Marital status*	Unmarried (Ref = Married)	0.371** (0.186,0.739)
	Others like, widowed/divorced/separated (Ref = Married)	0.674 (0.286,1.586)
*Occupation*	Labor (Ref = Agriculture worker)	1.827 (0.633,5.277)
	Sales worker (Ref = Agriculture worker)	1.836 (0.626,5.382)
	Service worker (Ref = Agriculture worker)	1.295 (0.412,4.071)
	Housewife (Ref = Agriculture worker)	1.337 (0.504,3.547)
	Transport worker (Ref = Agriculture worker)	1.527 (0.455,5.127)
	Small business (Ref = Agriculture worker)	2.056 (0.546,7.735)
	Not working/unemployed (Ref = Agriculture worker)	1.583 (0.572,4.385)
	Others (Ref = Agriculture worker)	0.230* (0.0545,0.974)
*Education level*	Primary level (Ref = No institutional education)	1.069 (0.697,1.639)
	Junior level (Ref = No institutional education)	1.169 (0.689,1.983)
	Secondary level (Ref = No institutional education)	1.084 (0.555,2.118)
	Higher Secondary level (Ref = No institutional education)	0.948 (0.361,2.487)
	Tertiary level and other (Ref = No institutional education)	0.766 (0.177,3.304)
*Location*	Urban (Ref = Rural)	0.686 (0.456,1.031)
*Illness or symptoms suffered*	Non-communicable diseases (Ref = Communicable diseases)	2.823*** (1.543,5.164)
	Accident and Injuries (Ref = Communicable diseases)	3.969** (1.468,10.73)
	Female reproductive health problem and delivery care (Ref = Communicable diseases)	6.204** (1.821,21.13)
	Symptoms (Ref = Communicable diseases)	0.493** (0.305,0.796)
	Others (Ref = Communicable diseases)	6.125*** (3.236,11.60)
*Inpatient care utilized*	Yes (Ref = No)	8.365*** (3.659,19.13)
*Household size*	4–5 persons (Ref = < = 3 persons)	0.877 (0.412,1.865)
	= >6 persons (Ref = < = 3 persons)	1.045 (0.492,2.220)
*Asset quintiles*	2nd (Ref = Poorest)	1.152 (0.635,2.088)
	3rd (Ref = Poorest)	2.424** (1.351,4.351)
	4th (Ref = Poorest)	3.721*** (1.996,6.937)
	Richest (Ref = Poorest)	6.954*** (3.580,13.51)
Constant		0.252 (0.0588,1.082)
N		1,601
LR chi2(32)		146.9
Prob > chi2		0.000

* p<0.05.

** p<0.01.

*** p<0.001.

## Discussion

The study showed that the utilization of MTP is higher among the insured group compared to the matched uninsured group in last three months. Multilevel logistic regression analysis demonstrated that the CBHI scheme beneficiaries were 2.111 times more likely to utilize MTPs. Healthcare from MTP became more accessible to the informal worker when they enrolled to the CBHI scheme. While UHC aims at increasing the number of population covered through risk pooling mechanisms (like, tax and insurance), covering informal workers poses a challenge. This pilot scheme shows how labor cooperatives may be used to bring more people under risk pooling mechanisms and indicates that people can benefit from access to better healthcare from MTPs. A separate study showed that this scheme enrolees spent lower OOP payments for such healthcare utilization compared to the matched uninsured group [29]. Through Chi-square test, we found that the reported illnesses were not associated with individuals’ insurance status (Table 3). Therefore, the CBHI scheme enrolment was not associated with moral hazard. Moral hazard implies that individuals utilize insurance more than they need in order to maximize their utility [30]. We performed a multiple logistic regression model analysis using self-reported illness or symptoms (1 = reported any illness or symptoms, 0 = reported none) as a dependent variable and individuals’ health insurance status as explanatory variable along with other control variables (S1 Table). In this analysis no significant association were observed between self reported illness or symptoms and individuals’ insurance status. However, the effect of CBHI scheme enrolment on health may not be clear due to ex-ante moral hazard [31,32] and endogeneity problem. Due to unavailability of baseline data we were unable to estimate lagged improvement in health because of CBHI scheme enrolment.

Though the CBHI scheme showed significant impact on increasing the utilization of MTP, a good number of members utilized untrained providers such as drug sellers (22%) and village doctors (23%). This may be because they are used to utilize this kind provider from historical use. Therefore it will take time to change their behaviour. Another influential factor may be that these types of provider are more ubiquitous in the community and travel time and travel costs for seeking them out are lower. Therefore, some logical modification in the CBHI scheme may be warranted. It is essential to conduct more frequent satellite clinics in the community to minimize these travel costs to members. Behaviour change communication intervention or educational intervention can be conducted for CBHI scheme members to teach them the importance of utilizing MTP. The medically trained healthcare workforce is scarce in Bangladesh, which is a challenge for the implementation of this kind of intervention [33]. However, in the long term, more trained healthcare workers may become available as the demand for such workers extended through scale-up of this intervention.

We found utilization of MTPs significantly less among unmarried household members (OR = 0.371; 95% CI: 0.186–0.774) than married members. The healthcare need may be higher among married members compared to unmarried members that resulted in higher MTP utilization by the married group. Similar finding was reported by earlier studies. Sultana et al. found that the health related quality of life (HRQoL) was higher among unmarried members (HRQoL score = 0.83) compared to married members (HRQoL score = 0.75) [34]. Another study showed that the married men were more likely to report an illness than unmarried [35]. Joung et al. 1995 found that utilization of healthcare facility were higher among the married (48.8%) compared to unmarried (43.8%) [36].

One possible limitation of this study was that we could not capture the seasonal variation in utilization of healthcare since the survey took place from April to June 2014. However, the use of a comparison group in the study from same community and the use of PSM during analysis could minimize such bias. While the basic variables were controlled and matched between the groups, there were still remaining important variables (e.g. travel time and cost) that could have caused the differences between the two groups. However, this is another potential limitation that we were unable to control for other unobserved factors.

There is a relatively small literature looking at the impact of CBHI on healthcare utilization [37]. Wagstaff et al. 2009, evaluated China’s cooperative medical scheme and found that it led to increased outpatient and inpatient utilization [26]. Gnawali et al. 2009 found a 40% increase in the utilization of outpatient visits among CBHI enrolees in Burkina Faso compared to a non-insured group [38]. A study conducted in Philippines reported higher utilization of hospitalization, consultation, diagnostic services among micro health insurance enrolees, though the study does not assessed separately the utilization of MTPs [18]. Mebratie et al. 2014 estimated CBHI scheme in Ethiopia lead to a 45% to 64% increase in utilization of outpatient services [39]. We found similar impacts in this study of CBHI scheme.

This scheme has potential to be scaled-up in existing cooperatives. Cooperatives in Bangladesh are organized under the Department of Cooperatives of the Government of Bangladesh. There are 1,107 central cooperatives with 133,188 members and 163,408 primary cooperatives with 8.5 Million members [40]. These cooperatives are not exclusively for informal workers. However, there are existing cooperatives of informal workers and there is large scope for such workers to initiate cooperatives for developing social protection along with their economic interest (micro-credit, trading, land owning etc.) where health can be incorporated as a strong component using insurance mechanism or mutual health organization. These new cooperatives can also be created for developing CBHI scheme only.

The healthcare financing strategy of Bangladesh emphasizes the importance of including informal workers into pre-payment schemes. Our experience suggests that CBHI is a platform for doing this. These are consequently established on the basis of common interest and solidarity among members and can be utilized as a platform for developing mutual health organization. Further research is required by offering different combinations of benefits (health insurance alone and/or savings and/or micro-credit and/or subsidy of food) for designing the schemes on the basis of more evidence. It is thus important to emphasis here that CBHI can be a valuable tool for achieving progress towards UHC.

## Conclusion

This study shows that the CBHI scheme for informal workers is likely to increase healthcare utilization from MTP. These types of schemes should be considered for scale up in other parts of the country as informal sector workers dominate the labor market of Bangladesh. Ensuring healthcare for informal sector workers (and their dependents) is a challenge for achieving UHC in many LMICs and CBHI schemes can potentially address this challenge. However, further studies are required to understandthe potential strengths and limitations of implementing this kind of scheme in low income settings. Further, a cost-benefit analysis can be conducted to observe the economic feasibility of this scheme before scaling up.

## Supporting information

S1 Table Association between self-reported illness or symptoms and individuals’ health insurance status. (PDF)

S1 Dataset (DTA)

S1 Questionnaire (PDF)

S2 Questionnaire (PDF)
