# Supplementary material for: The impact of community-based health insurance on the utilization of medically trained healthcare providers among informal workers in Bangladesh
Source: PLoS One. 2018 Jul 11;13(7):e0200265. doi: 10.1371/journal.pone.0200265 (PMC6040718; doi:10.1371/journal.pone.0200265)
Supplement: S1 Table — (PDF) [file pone.0200265.s001.pdf]

S1 Table. Association between self-reported illness or symptoms and individuals' health insurance status

Characteristics	Description	Dependent variable = Self reported illness or symptoms (1= reported any illness or symptoms, 0= reported none)
Health insurance status	Member (Ref= No membership)	1.109(0.982,1.252)
Age-group	Adult, 15-60 years (Ref= Child, 0-14 years)	0.700**(0.550,0.891)
	Elderly, 60+ (Ref= Child, 0-14 years)	0.739(0.524,1.042)
Gender	Female (Ref= Male)	1.111(0.935,1.320)
Marital status	Unmarried (Ref=Married)	0.475***(0.378,0.597)
	Widowed/divorced (Ref=Married)	0.884(0.622,1.258)
Occupation	Labor (Ref= Agriculture worker)	0.523**(0.341,0.802)
	Sales worker (Ref= Agriculture worker)	0.627*(0.403,0.977)
	Service worker (Ref= Agriculture worker)	0.397***(0.252,0.625)
	Housewife (Ref= Agriculture worker)	0.824(0.548,1.238)
	Transport worker (Ref= Agriculture worker)	0.625(0.387,1.010)
	Small business (Ref= Agriculture worker)	0.663(0.389,1.130)
	Not working/unemployed (Ref= Agriculture worker)	0.646*(0.427,0.978)
	Others (Ref= Agriculture worker)	0.739(0.417,1.309)
Education	Primary level (Ref= No institutional education)	0.817*(0.692,0.966)
	Junior level (Ref= No institutional education)	0.627***(0.516,0.761)
	Secondary level (Ref= No institutional education)	0.577***(0.453,0.737)
	Higher Secondary level (Ref= No institutional education)	0.922(0.633,1.344)
	Tertiary level and other (Ref= No institutional education)	0.769(0.452,1.310)
Income quintiles	2nd (Ref=Poorest)	0.914(0.753,1.111)
	3rd (Ref=Poorest)	0.963(0.793,1.170)
	4th (Ref=Poorest)	1.007(0.824,1.231)
	Richest (Ref=Poorest)	0.857(0.702,1.046)
Household size	4-5 persons (Ref= <=3 persons)	0.756(0.566,1.009)
	=>6 persons (Ref= <=3 persons)	0.809(0.606,1.080)
Location	Urban (Ref=Rural)	1.474***(1.288,1.687)
Constant		1.375(0.793,2.383)
N		5,038
LR chi2(32)		241.5
Prob > chi2		0.000
