# Supplementary material for: The impact of community-based health insurance on the utilization of medically trained healthcare providers among informal workers in Bangladesh
Source: PLoS One. 2018 Jul 11;13(7):e0200265. doi: 10.1371/journal.pone.0200265 (PMC6040718; doi:10.1371/journal.pone.0200265)
Supplement: S1 Questionnaire — (PDF) [file pone.0200265.s003.pdf]

Study title: Self-financed health scheme of labour cooperative for accessing quality healthcare of informal sector workers – An implementation research on an existing cooperative for social protection.

Section 1A: Identification information
(Should be removed after completing data-entry & cleaning process)

Questionnaire No:

Name of respondent: ____

Respondent did House Hold Head, 1. Yes, 2. No

Respondents Address: ____

____

Phone number: |___|___|___|___|___|___|___|___|___|___|___|

Phone number: 1. Own 2. Other (please specify ___________________________)

LASP members: ____

Member Regi. ID: |___|___|___|___|___|___|___|___|___|___|___|

Date of LASP entry | ___ | ___ |: | ___ | ___ |: | ___ | ___ | ___ | ___ | (Day / Month / Year)

Interview of Interviewer code: | ___ | ___ |

Interview start time: |___|___|: |___|___| ☐ am/ ☐ pm

Interview end time: |___|___|: |___|___| ☐ am/ ☐ pm

Date of Interview | ___ | ___ |: | ___ | ___ |: | ___ | ___ | ___ | ___ | (Day / Month / Year)

Section 1B: Consent form

Study title: Self-financed health scheme of labour cooperative for accessing quality healthcare of informal sector workers – An implementation research on an existing cooperative for social protection.

Principal Investigator (PI): Dr. Jahangir A.M. Khan
Organization: icddr,b

Questionnaire ID:

Greetings. I (Name of the interviewer) ---------------- icddr,b, a international health research organization .We are conducting a survey in this area for informal workers. The purpose of this research is to assess impact of ongoing health insurance scheme on affordability, equity and utilization of healthcare among member informal workers compared to non-member informal workers. For this purpose we ask you about your income, asset, healthcare utilization and related expenses.

Why did we select you?
Since this project is being conducted in this area and you are randomly selected for this study, we consider you as a respondent of this study.

Method:
If you agree to participate in this research study, it would involve an interview lasting about 50 minutes. In this interview, I shall ask you about your income and your time involvement and expenditure to receive this service. You can choose your comfortable place for interview. If you agree I can start interview now or I can come again at your convenient time.

Privacy, anonymity and confidentiality:
We are assuring you that information given by you will be kept strictly confidential. We also want to assure you that all paper records of the interviews will be kept in a safe and secure place for five years and will not be used for any other purpose than the study. We ensure you that your name and other identity will not be exposed while the research results will be published. So it will not be possible to trace the answers back to you.

Future use of information:
Information provided by you will be used for this research only and your name will not be exposed when the research results will be published.

Risk:
There are no physical and social risks related to your participation in this study and if you refuse to participate in this study, you and your family will not faces any risk.

Benefit:
You will not be directly benefited by participating in this study. However**,** this information will improve the efficiency and quality of this service.

***Freedom not to participate and withdraw*:**
You are absolutely free to either participate or not participate in the study. You are free not to answer any question if you wish. Even you are free to withdraw at any point of the interview.

Compensation:
The study is unable to provide any financial compensation to you.

If you have any query regarding the study, you are free to ask the interviewer. You can also contact the principal investigator of this study or IRB coordinator at the address given below.

Dr. Jahangir A. M. Khan
Principal Investigator,
icddr,b,
Mohakhali, Dhaka-1212
Telephone: 02-8860523-32 (Ext.2531)
or 02-8811155 (Direct)
Email: Jahangir.khan@icddrb.org

M. A. Salam Khan
IRB coordinator,
Research Administration Services,
icddr,b
Mohakhali, Dhaka-1212
Telephone:02-8860523-32 (Ext. 3206)
Email: salamk@icddrb.org

Are you agree to participate in this study Yes ☐ No ☐

______________________________ ______________
Signature or left thumb impression of participant Date

______________________________ ______________
Signature of the Interviewer Date

Section 2 : Information about Household

2.1	What is the interval of household head income ?<br>How much does he earn?	□ 1. Daily<br>□ 2. Weekly Income taka ________________________________<br>□ 3. Monthly (skip 2.2)
2.2	If the earning is daily or weekly basis, how many days did the household head work in the last three months?	First month _________________________________days<br>Second month _________________________________ days<br>Third month _________________________________ days
2.3	What are the sources of household head income in the past 12 months? (More than one answer may be)	(see table below)

Sl.No	Source of income	Yes	No
1	Agriculture (Own)	1	2
2	Share-farming	1	2
3	Mortgage	1	2
4	Daily labor	1	2
5	Fishing / Sell	1	2
6	Cattle / poultry / duck farms	1	2
7	Crafting	1	2
8	Tailor	1	2
9	Business	1	2
10	Employee	1	2
11	Retirement allowance	1	2
12	Cash received (internal)	1	2
13	Cash received (foreign)	1	2
14	food for work	1	2
15	Distribute various / old age pension / allowance	1	2
16	Home/ Shop rent	1	2
17	Riksha/ Van/ Auto driver	1	2
18	Others (Specify) ______________	1	2

2.4	Of the mentioned sources above (2.4), what was the main source of income?	Number |___|___| (Enter the serial number of the question 2.4)
2.5	How many rooms are there in the house?	Number: |___|
2.6	What are the construction materials of the rooms (to verify)?	1. Pucca / semi-pucca<br>2. Tin<br>3. Tin & bamboo<br>4. Tin & others<br>5. Bamboo & others<br>6. Raw (soil)<br>7. Wood<br>8. Ceramic / Tiles<br>9. Others

	Ceiling	Wall	Floor
Sl. No			
8 (eight) to specify			

2.7	How much land do your family have?	Homestead (including ponds and puddles):<br>Kani: _______ Gonda: _______ Kora:_______= Decimal |___|___|___|___| Agriculture land:<br>Kani_______ Gonda_______ Kora_______ = Decimal |___|___|___|___|
2.8	Specific goods / assets exist in your household (More than one answer may be)	(see table below)

**No.**	**items/**	Yes	No
1	Cow / goat	1	2
2	Fishing net	1	2
3	duck / Chicken	1	2
4	Grocery	1	2
5	Riksha/Van/ Auto Riksha	1	2
6	Agticultural instruments	1	2
7	Boat	1	2
8	Bed	1	2
9	blanket	1	2
10	Quilt	1	2
11	Hurricane	1	2
12	Chair/ Table	1	2
13	Dining Table	1	2
14	Almirah / Showcase	1	2
15	Sofa Set	1	2
16	Television	1	2
17	Radio/ Tap Recorder	1	2
18	Clock / wall clock	1	2
19	Telephone/ Mobile Phone	1	2
20	Bicycle	1	2
21	Motorcycle	1	2
22	Freeze	1	2
23	Fan	1	2
24	Sewing-machine	1	2
25	Others (Specify) ______________________________	1	2

2.9	What type of toilet do you use?	(see table below)

SL. No	Toilet type	Yes	No
1	Sanitary	1	2
2	Pit Toilet	1	2
3	Paka Toilet	1	2
4	Raw Toilet	1	2
5	Others (Specify)______________________	1	2

2.10	What is the main source of your drinking water?	(see table below)

**Sl** No	Source type	Yes	No
1	Tube well	1	2
2	Ponds / rivers / puddles / streams	1	2
3	Pure / filtered water	1	2
4	Rain Water	1	2
5	Pipe / tap / Supply Water	1	2
6	Others (Specify)____________________	1	2

Section 3: Information about household members

Col-1	Col-2	Col-3	Col-4	Col-5	Col-6	Col-7	Col-8	Col-9	Col-10	Col-11	Col-12			
Member code	Name (Short name)	Relationship with the Household head	Sex	Age (Fullyear)	Marital status	Educational Qualification*	Main occupation	How many days did you work in the last month?	Monthly Income (Taka)	LASP service giver	Any member of an NGO, association, or insurance members / beneficiaries?			
		1 Self<br>2 Husband/wife<br>3 Brother/Sister<br>4 Son / Daughter /Son-in-law / daughter-in-law<br>5 Mother / father / father-in-law / mother-in-law<br>6 Grandfather / grandmother<br>7 Grandson / granddaughter<br>8 Aunt<br>9 Non-relative<br>10 Others (Specify)	1 Male<br>2 Female		1 Married<br>2 Unmarried<br>3 Widower/Widow<br>4 Divorced<br>5 Separated		1 Labor<br>2 Rickshaw/ Van<br>3 Auto/ CNG driver<br>4 Resturent staff<br>5 Shopkeeper<br>6 Garments staff<br>7 Tailor<br>8 Barber<br>9 Grosser/ tea shop<br>10 Mobile Business<br>11 Employee<br>12 Farm<br>13 Share-farming<br>14 Fisherman<br>15 Small Cottage Industry<br>16 Daily Labor<br>17 Mechanic / Electrician<br>18 Housekeeper<br>19 Housewife<br>20 Unemployed<br>21 Disability<br>22 Student<br>23 Child / Adult<br>24 Others (Specify)			1 Covered<br>2 Not covered	1. Yes<br>2. No	1 Grameen Bank<br>2 BRAC<br>3 ASA<br>4 SAIEF<br>5 TMSS<br>6 Insurance<br>7 Others (Specify)<br><br>NGO or Co-operative		

Code illiterate / institutional education has (1) , primary / PSC unfinished (II) , primary / PSC ends (3) , eighth-grade / JSC unfinished (4) , eighth-grade / JSC ends (5) , Secondary / submitted incomplete (6) , Intermediate / submit completed (7) , higher secondary / Aleem unfinished (8) , higher secondary / Aleem ends (9) , bachelor / honors / Fajel unfinished (10) , bachelor / honors / Fajel finished (11) , Masters degree / Kamil unfinished (1) , a Masters degree / Kamil ends (13)

**Section 4:** Information about the households expenditure				
**Part A: Food (weekly consumption)**				
Col-1	Col-2	Col-3	Col-4	Col-5
Sl. No	Types of food	Total consumption/ food intake	**Cost (market price)**	Source (1=Purchase, 2=Exchange to work, 3=own production, 4= Gift
**4.1** food intake in last week				
1	Rice (kg)			
2	Wheat(kg)			
3	Flour (kg)			
4	Cira/ Muri			
**4.2** pulses intake in last week				
5	Lentil (kg)			
6	Bengal gram pulses (Kg)			
7	Mush Kalai (Kg)			
8	Pea gram (Kg)			
9	Chickling-Vetch (Kg)			
10	Green gram (Kg)			
11	Anchor (Kg)			
**4.3** Edible oil in the last one week				
12	Soabin (Kg)			
13	Mustard (Kg)			
14	Dalda (Kg)			
15	Ghee (Kg)			
16	Palm Oil (Kg)			
17	Others (Specify)_________________________			
**4.4** Greens / vegetables intake in last week				
18	Red leafy (Kg)			
19	Pui leafy (Kg)			
20	Spinach (Kg)			
21	Spinach ipomoea aquatic(Kg)			
22	Others leafy _________________ (Kg)			
23	Others leafy _________________(Kg)			
24	Others leafy _________________(Kg)			
25	Potato (Kg)			
26	Cataract (Kg)			
27	Gourd (Kg)			
28	Cauliflower			
29	Cabbage			
30	Brinjal (Kg)			
31	Arum			
32	Gourd			
33	Tomato (Kg)			
34	Green papaya(Kg)			
35	Green banana (Kg)			
36	Chili (Kg)			
37	Onion (Kg)			
38	Garlic (kg)			
39	Ginger (kg)			
40	Others vegetables (Specify)_________________(kg)			
41	Others vegetables (Specify)_________________(kg)			
42	Others vegetables (Specify)_________________(kg)			
**Part B: Food (Monthly consumption)**				
**4.5** Spice last month				
1	Dried chili (gm)			
2	Turmeric (gm)			
3	Cumin (gm)			
4	Cinnamon (gm)			
5	Cardamom (gm)			
6	Others spice __________________(gm)			
7	Others spice __________________(gm)			
8	Others spice __________________(gm)			
**4.6** Fish, meat, eggs and milk intake in last month				
9	Large Fish (kg)			
10	Smaller Fish (kg)			
11	Dried fish (kg)			
12	Beef (kg)			
13	Buffalo (kg)			
14	Local chicken meat (kg)			
Sl.No	Food types	Total consumption/ Food intake	**Consumption (Market price) in BDT**	Source (1= Purchase, 2=Exchange to work, 3=own production, 4=Gift)
15	Farm chicken meat (kg)			
16	Duck (kg)			
17	Mutton (kg)			
18	Egg (number)			
19	Milk (Kg)			
20	Other non-vegetarian foods___________(Kg)			
21	Other non-vegetarian foods ___________(Kg)			
22	Other non-vegetarian foods ___________(Kg)			
**4.7** Fruits intack in last one month				

23	Banana (number)			
24	Boroy (Kg)			
25	Olive (kg)			
26	Ripe papaya (kg)			
27	Mango (kg)			
28	Jack kfruit (number)			
29	Blackberry (kg)			
30	Apple (kg)			
31	Orange (kg)			
32	Grapes			
33	Others fruits _______________ (kg)			
34	Others fruits _______________(kg)			
35	Others fruits _______________(kg)			
**4.8** Others food intack in last month				
36	Sugar (kg)			
37	Molasses (kg)			
38	Powder milk (kg)			
39	Salt (kg)			
40	Noodles (kg)			
41	Suji/ Semai (kg)			
42	Soft drinks (ltr)			
43	Sweet types food (kg)			
44	Child food _________________(kg)			
45	Tea leaves (kg)			
46	Biscut (kg)			
47	*Chanachur* (kg)			
48	Others (Specify)_______________(kg)			
49	Others (Specify)_______________(kg)			
50	Others (Specify)_______________ (kg)			
**Part C: Tobacco products (Weekly expense)**				
**4.9** Tobacco intact in last week				
1	Batel leaf (number)			
2	Betel nut (number)			
3	Tobbacco leaf (Kouta)			
4	Tobbacco (number)			
5	Gul (Kouta)			
6	Biri (Packet)			
7	Cigaret (Packet)			
8	Others (Specify)_______________ (kg)			
9	Others (Specify)_______________(kg)			
10	Others (Specify)_______________(kg)			

Part D: Non food (Monthly expenditure)

Col-1	Col-2	Col-3
**Sl. no**	**Types**	Cost amount (monthly)
**4.10** Non-food expenditure in last month		
1	Rent	
2	Electricity Bill / Solar Power	
3	Gas bill	
4	Fuel wood	
5	Fuel oil (kerosene, petrol ...)	
6	Transport	
7	Health	
8	Education (school fees, coaching, private teacher, etc.)	

Col-1	Col-2	Col-3
**Sl.No**	**Types**	Cost amount (monthly)
9	Notebooks / Pens / books / other study materials	
10	Communication expense (mobile, telephone, etc.)	
11	Dish bills / internet bill	
12	Cosmetics expense (Cream, Powder, make-up etc.)	
13	Hair Oil (Narikel Oil)	
14	Cleaning and hygiene (soap, detergent, shampoo etc.)	
15	Laundry	
16	Money expense (any type)	
17	Co-operative instalment / Micro-credit instalment	
18	Salon / Spa	
19	Others (Specify)	
	**Part C: Non food (Yearly expenditure)**	
**4.11 Non food expenditure in last year**		
1	Clothing	
2	Shoe	
3	Quilt / quilt / pillow / blanket / bed	
4	Entertainment / religious festival	
5	House construction / House Renovation	
6	Furniture	
7	Insurance Premium	
8	House keeper/ maids salary	
9	Gifts provide	
10	Donation	
11	Travel	
12	Others (Specify)	

Section 5: Information about LASP services

**5.1**	Taking LASP services in last three months				
	**Services**	**Services recever code**	**Amount of service**	**Total cost of service giver**	**Elsewhere to receive the same service cost / market price**
1	Suggestions of MBBS doctor				
2	Service of Satellite clinic				
3	Service of Health camp				
4	Service of specialist doctor				
5	Diagnostic services				
6	Allowance of hospitalized medical services				
7	Allowance of Drug				
8	Others health services________				
9	Computer training				
10.	Services of LASP store	**Products**	**Amounts**	**Total cost of service giver**	Elsewhere on the value of the product / market price
		Rice			
		**Peas**			
		**Flour**			
		**Sugar**			
		**Soybean**			

**5.2**	If you have not received LASP service and causes		
**1. Services**	☐ **Did not accept health services**	☐ **Did not accept computer service in family member**	☐ **Product of LASP store**
2. Cause of deny of services	1. Any member of the family was not sick	1. did not have any eligible children of the family	1. No LASP store hear in your area
	2. Was sick, but the complex was not any problems	2. Any member of your family is not interested	2. Could not credit sale in LASP Store
	3. LASP medical expenses too much	3. Excessive training costs	3. Credit purchase in other store
	4. Was not a sufficient amount of money for LASP medical services	4. Was not a sufficient amount of money for LASP training	4. LASP store cannot credit sale
	5. very much distance the LASP health centre	5. Was not informed the trainging services	5. Far distance of LASP centre
	6. Nobody here to take LASP centre	6. The training is suitable at least for the next	6. LASP store have not sufficent product
	7. Didn't know about the location about the LASP centre	7. very far distance for LASP centre	7. Not know about the LASP store
	8. LASP service didn't good	8. LASP training quality was not fine	8. Not good quality of product in LASP centre
	9. Others (Specify)	9 Others (Specify_________________________)	9 Others (Specify)_________________)

Section 6: Household information about illness in last three months

Do you or any member of your households in the last three months, with no signs of disease / pregnancy problems / child birth, what was? □ Yes(1), □ No (2) (If yes, please fill in the following table)

Col-1	Col-2	Col-3	Col-4	Col-5	Col-6		Col-7	Col-8	Col-9										Col-10	Col-11					
(Member Code)	Enter the code of the member *	How much days the individual suffered from this disease?	Did you seek any health care for those diseases? Code 1 = Yes, 2= No (If the answer is not to go to the next	If you did not seek care, why?<br>1. Not any complicated problem<br>2. Very costly treatment<br>3. Not enough money<br>4. Far distance of health care<br>5. There was none to accompany<br>6. Didn't know where to go<br>7. Quality of healthcare is not good<br>8. Other, specify	If yes, from where you got treatment?<br>1.Village Doctor<br>2. Drug-seller<br>3. MBBS doctor<br>4. Community clinic<br>5. NGO clinic<br>6. Private clinic<br>7. Upazila Health Complex<br>8. Medical Hospital / District<br>9. LASP centre<br>10. LASP health/ Satelite Cam<br>11.Self-treatment<br>12. Traditional healer<br>13. Other, specify		Did you get admitted to hospital due to the problem or disease? Code: 1= Yes, 2= No	If yes, how long you had to stay at hospital? (Daylong)	Treatment cost<br>(Does not apply to the cost, if '0 'Enter')										How long absent due to illness? (Full day)	How did you bear the cost of treatment?<br>1. Regular income<br>2. family savings<br>3. Health Insurance<br>4. Except on health insurance<br>5. Selling goods<br>6. Sold on Trees / agricultural / livestock animals<br>7. Fixed assets sold<br>8. Mortgage of land or property<br>9. Borrow money from local merchants<br>10. With help from friends or relatives<br>11. Relatives / friends / colleagues borrow from<br>12. Any organization / association borrowings fro<br>13Other, specify					
					SL no	Source of treatment			Consultiation	Drug	Bed cost	Daignostic cost	transpot	Tips	Attendent cost	Package Cost	Other	Total cost		Source-1	Taka	Source-2	Taka	Source-3	Taka
					1.																				
					2.																				
					3.																				
					1.																				
					2.																				
					3.																				
					1.																				
					2.																				
					3.																				
					1.																				
					2.																				
					3.																				
					1.																				
					2.																				
					3.																				

* 1 = cold, cough, fever 2 =, 3 = Typhoid, 4 = diarrhea / diarrhea / stomach, 5 = up, 6 = asthma / shortness of breath, 7 = primary injury, 8 = boon fracture ,9 = jaundice, 10 = weak, 11 = worms, 12 = headache, 13 = skin, convulsions = 14, 15 = chest pain, 16 = high blood pressure, 17= diabetes, 18 = tuberculosis, 19 = silver, 20 = palsy / paralysis, 21 = arthritis, 22 = rheumatic fever, 23 = cancer, 24 = child labor, 25 = Pregnancy problems 26 = Mental problems, 27 = other (please specify)

**Section 7: Impact of** illness of working members on household<br>(If LASP member did not suffer illness finish interview)			
**Part 7A:** While the presence of illness			
1	During the past 3 months, how many days the LASP members had to work despite of illness? (If not applicable, "NA" Enter)		
**Part 7B:** Drop - out from work due to illness,			
2	Did any of the LASP members permanently / temporarily left the work due to illness in the last 3 months?	☐ (1) Yes<br>☐ (2) No<br>☐ (99) Not Applicable	
**Part 7C:** The impact of illness on food security			
3	Did your family members have to reduce the food and other consumption to cover the treatment cost in the last 3 months?	☐ (1) Yes<br>☐ (2) No<br>☐ (99) Not Applicable	
**Part 7 D:** Children drop out of school due to illness			
4	In the past 3 months, did any of the children drop out from the school due to illness?	☐ (1) Yes<br>☐ (2) No<br>☐ (99) Not Applicable	Member code
**Part 7 E:** Child labor due to illness			
5	In the last 3 months, did any of child members of your household have to work due to your illness?	☐ (1) Yes<br>☐ (2) No<br>☐ (99) Not Applicable	Member code
**Section 8:** Satisfaction on health services<br>(LASP member who get LASP health services in past three month will respond this section)			
1	What is your opinion about the doctor's behaviour of health centre?	1. Very bad<br>2. Bad<br>3. Fairly<br>4. Good<br>5. Very good	
2	Did the doctors provide you clear instruction on taking medicine and advice?	1. Not be explained simply<br>2. Partly explained<br>3. Fairly explained<br>4. Has been explained<br>5. Been fully explained	
3	Did the doctor clearly explain to you about the prescribed test?	1. Not be explained simply<br>2. Partly explained<br>3. Fairly explained<br>4. Has been explained<br>5. Been fully explained	
4	What is your opinion about the behaviour of other staff?	1. Not be explained simply<br>2. Partly explained<br>3. Fairly explained<br>4. Has been explained<br>5. Been fully explained	
5	Reception rooms, toilets and other materials were clean?	1. Was very untidy<br>2. Scruffy was<br>3. Were fairly clean<br>4. Clean<br>5. Very clean	
6	What is your overall feeling about the services of the facility?	1. Very bad<br>2. Bad<br>3. Fairly<br>4. Good<br>5. Very good	

Thank you for giving your valuable time
