# Supplementary material for: The impact of community-based health insurance on the utilization of medically trained healthcare providers among informal workers in Bangladesh
Source: PLoS One. 2018 Jul 11;13(7):e0200265. doi: 10.1371/journal.pone.0200265 (PMC6040718; doi:10.1371/journal.pone.0200265)
Supplement: S2 Questionnaire — (PDF) [file pone.0200265.s004.pdf]

Study title: Self-financed health scheme of labour cooperative for accessing quality healthcare of informal sector workers – An implementation research on an existing cooperative for social protection.

**Section 1A: Identification information**<br>(Should be removed after completing data-entry & cleaning process)
**Questionnaire No:**
উত্তরদাতার নাম: ____________________<br>উত্তরদাতা কি খানাপ্রধান, 1. হ্যাঁ 2. না<br>উত্তরদাতার ঠিকানা: ____________________<br>____________________<br>ফোন নাম্বারঃ |___|___|___|___|___|___|___|___|___|___|___| ফোন নম্বর 1. নিজের 2. অন্যের (উল্লেখ করুন ____________)
ল্যাম্প সদস্যের নাম: ____________________<br>সদস্যের Regi. ID: |___|___|___|___|___|___|___|___|___|___|___| ল্যাম্পে সদস্যভুক্তির তারিখঃ |___|___|:|___|___|:|___|___|___|___| (দিন/মাস/বছর)
সাক্ষাৎকার গ্রহণকারীর কোডঃ |___|___| সাক্ষাৎকার গ্রহণ শুরুর সময়ঃ |___|___|: |___|___| ☐ am/ ☐ pm<br>সাক্ষাৎকার গ্রহণ শেষের সময়ঃ |___|___|: |___|___| ☐ am/ ☐ pm<br>সাক্ষাৎকার গ্রহণের তারিখঃ |___|___|:|___|___|:|___|___|___|___| (দিন/মাস/বছর)

Section 1B: Consent form

Study title: Self-financed health scheme of labour cooperative for accessing quality healthcare of informal sector workers – An implementation research on an existing cooperative for social protection.

Principal Investigator (PI): Dr. Jahangir A.M. Khan
Organization: icddr,b

Questionnaire ID:

গবেষণার উদ্দেশ্যঃ
আসসালামু আলাইকুম / আদাব। আমি (তথ্য সংগ্রকারীর নাম) ----------------- আইসিডিডিআর, বি (কলেরা হাসপাতাল) নামক আন্তর্জাতিক একটি গবেষণা প্রতিষ্ঠান হতে এসেছি। আমরা এই এলাকায় অনৈয়মিক শ্রমিকদের জন্য সমবায় সমিতির মাধ্যমে কিভাবে টেকসই মানসম্মত স্বাস্থ্যসেবা দেয়া যায় সে সম্পর্কিত একটি গবেষণা পরিচালনা করছি। আমাদের এই গবেষণার উদ্দেশ্য হল যে অনৈয়মিক শ্রমিকদের নিজস্ব অর্থায়নে সমিতির মাধ্যমে স্বাস্থ্যসেবার সুযোগ এবং এর উপযুক্ত ব্যবহার নিশ্চিত করা যায় এ সম্পর্কিত প্রকল্পটির মূল্যায়ন করা, স্বাস্থ্যসেবা নিতে নিজের কত টাকা ব্যয় হয় তা নির্ণয় করা। এই জন্য আমরা আপনাকে আপনার আয় , স্বাস্থ্যসেবার সুযোগ এবং এর উপযুক্ত ব্যবহার ,স্বাস্থ্যসেবা নিতে নিজের ক ত টাকা ব্যয় হয় এবং কিভাবে এই অর্থের যোগান হয় সে সম্পর্কিত কিছু প্রশ্নাবলী জিজ্ঞেস করবো।

আপনাকে কেন এই গবেষণায় অন্তর্ভুক্ত করা হলো?
যেহেতু এখানে আমাদের প্রকল্পটি চালু আছে এবং র্যাব নডোম নমুনা নির্বাচন প্রক্রিয়ায় আপনি নির্বাচিত হয়েছেন , সেহেতু আপনাকে এই গবেষণায় একজন উত্তরদাতা হিসাবে নির্বাচিত করা হয়েছে।

পদ্ধতিঃ
আপনি যদি আমাদের এই গবেষণায় অংশ নিতে আগ্রহী হন তবে ৫০ মিনিট স্থায়ী আপনার একটি সাক্ষাতকার নিব। এই সাক্ষাতকারে আমি আপনাকে আপনার আয় এবং এ প্রকল্পে সেবা গ্রহনে আপনার প্রদেয় সময় ও খরচ সম্প র্কিত বিষয়ে কিছু প্রশ্ন করবো। আপনি আপনার পছন্দনীয় স্থানে আপনি সাক্ষাতকার প্রদান করতে পারেন। আপনি চাইলে এখনি সাক্ষাতকার প্রদান করতে পারেন অথবা আপনার সুবিধাজনক সময়ে আমরা পুনরায় আসতে পারি।

তথ্যের গোপনীয়তা ও নিরাপত্তা
আপনার দেওয়া সকল তথ্যের গোপনীয়তা দৃঢ়ভাবে রক্ষা করা হবে। আমরা আরও আশ্বস্ত করতে চাই যে , সাক্ষাত প্রদানকারীর প্রদেয় সব তথ্য, ধারণকৃত তথ্যাবলী কাগজপত্র পাঁচ বছরের জন্য নিরাপদ ও গোপনে রাখা হবে যা কেবল মাত্র এই গবেষণায় ব্যবহার করা হবে। আপনার নাম এবং এমন কোন তথ্য যা আপনাকে সনাক্ত করতে পারে তা ভবিষ্যত গবেষণা প্রকাশনা হতে বাদ দেয়া হবে। তাই এটা কোন ক্রমেই সনাক্ত করা সম্ভব নয় যে এটা আপনার দেয়া উত্তর।

ভবিষ্যতে তথ্যর ব্যবহার
আপনার দেয়া তথ্য শুধু এই গবেষণার কাজে ব্যবহৃত হবে এবং গবেষণার ফলাফল প্রকাশের সময় আপনার নাম কোনভাবেই প্রকাশ করা হবে না।

ঝুঁকি
এই গবেষণায় অংশ গ্রহন করলে আপনি কোন শারীরিক বা সামাজিক ঝুঁকির সম্মুখীন হবেন না। এই গবেষণায় অংশ গ্রহন করতে রাজি না হলেও আপনি বা আপনার পরিবার কোন ধরনের সমস্যার সম্মুখীন হবে না।

লাভ
এই গবেষণায় অংশ গ্রহনের মাধ্যমে আপনি সরাসরি কোন লাভবান হবেন না কিন্তু ভবিষ্যতে প্রকল্পটির উপযোগীটা ও সেবার গুনগত মান বৃদ্ধিতে সাহায্য করবে।

গবেষণায় অংশ না নেওয়া এবং বর্জনের অধিকার
এই গবেষণায় অংশগ্রহন সম্পূর্ণ আপনার ঐচ্ছিক । যে কোন প্রশ্নের উত্তর না দেওয়া সম্পূর্ণ আপনার ইচ্ছাধীন। সাক্ষাতকারের যে কোন পর্যায়ে আপনি নিজেকে প্রতাহার করতে পারবেন।

ক্ষতিপূরণঃ
এই গবেষণা সম্বন্ধে আপনার কোন প্রশ্ন থাকলে আপনি গবেষণা কর্মীর কাছ থেকে জানতে পারবেন। আপনি প্রধান গবেষক অথবা আই , আর, বি, কো-অর্ডিনেটরের সাথেও নিম্নে দেওয়া ঠিকানায় যোগাযোগ করতে পারবেন।

ডঃ জাহাঙ্গীর এ.এম. খান
প্রধান গবেষক
আইসিডিডিআর, বি
মহাখালি, ঢাকা-১২১২
ফোন - ০২-৮৮৬০৫২৩-৩২ Ext :২৫৩১
অথবা ০২-৮৮১১১৫৫ (সরাসরি)
Email: jahangir.khan@icddrb.org

এম. এ. সালাম খান
আই. আর. বি. কো-অর্ডিনেটর
রিসার্চ এডমিনিস্ট্রেশন
আইসিডিডিআর,বি
মহাখালি, ঢাকা-১২১২
ফোন - ০২-৮৮৬০৫২৩-৩২ Ext :৩২০৬
Email: salamk@icddrb.org

আপনি কি এই গবেষণায় অংশ গ্রহনে রাজী? ☐ হ্যাঁ ☐ না

সাক্ষাতকার প্রদানকারীর স্বাক্ষর অথবা টিপ সহি ____________তারিখ _____________

সাক্ষাতকার গ্রহণকারীর স্বাক্ষর _______________________তারিখ _____________

Section 2 : সমগ্র খানা সম্পর্কিত তথ্য

2.1	খানাপ্রধান আয় কিভাবে পান? এবং কত পান?	□ 1. দৈনিক<br>□ 2. সাপ্তাহিক আয় টাকায়______________________<br>□ 3. মাসিক (2.3 এ যান)
2.2	যদি আয় দৈনিক অথবা সাপ্তাহিক হয় তবে গত তিন মাসে খানাপ্রধান কতদিন কাজ করেছিলেন?	প্রথম মাস ______________________ দিন<br>দ্বিতীয় মাস ______________________ দিন<br>তৃতীয় মাস ______________________ দিন
2.3	গত 12 মাসে খানাপ্রধানের আয়ের উৎসগুলি কি কি ছিল? (একাধিক উত্তর হতে পারে)	(see table below)

নং	আয়ের উৎস	হ্যাঁ	না
1	কৃষি (নিজস্ব জমি)	1	2
2	কৃষি (বর্গাচাষ)	1	2
3	বন্ধক/ কট/ পোষানি (দেওয়া নেওয়া)	1	2
4	দিন মুজুর	1	2
5	মাছ ধরা / বিক্রয় করা	1	2
6	গরু ছাগল/ মুরগী/ হাঁসের খামার	1	2
7	হস্তশিল্প	1	2
8	দর্জি কাজ	1	2
9	ব্যবসা	1	2
10	চাকুরী	1	2
11	অবসর ভাতা	1	2
12	নগদ অর্থপ্রাপ্তী (অভ্যন্তরীন)	1	2
13	নগদ অর্থ প্রাপ্তী (বিদেশ হতে)	1	2
14	কাজের বিনিময়ে খাদ্য	1	2
15	ভিজিডি/ বয়স্ক ভাতা/ দুস্থঃ ভাতা	1	2
16	বাড়ি/ দোকান ভাড়া	1	2
17	রিকশা/ভ্যান/অটো চালক		
18	অন্যান্য (উল্লেখ করুন) _______________	1	2

2.4	উপরে উল্লেখিত(2.4) আয়ের উৎসগুলির মধ্যে প্রধান উৎসটি কি ছিল?	নং |___|___| (2.4 নং প্রশ্ন হতে শুধু ক্রমিক নং লিখুন)
2.5	খানায় কতগুলি ঘর আছে?	সংখ্যাঃ |___|
2.6	প্রধান ঘর কি কি উপকরণ দ্বারা তৈরী (যাচাই করুন)	1. পাকা/ আধা পাকা<br>2. টিন<br>3. টিন এবং বাঁশ<br>4. টিন এবং অন্যান্য<br>5. বাঁশ এবং অন্যান্য<br>6. কাঁচা (মাটি)<br>7. কাঠ<br>8. সিরামিক/টাইলস<br>9. অন্যান্য

	ছাদ	দেওয়াল	মেঝে
ক্রমিক নং			
8 (আট) হলে উল্লেখ করুন			

2.7	আপনাদের পরিবারের কতটুকু জমি আছে ?	বসত ভিটা(পুকুর এবং ডোবা সহ):<br>কানিঃ______ গণ্ডাঃ______কড়াঃ______= শতাংশ|___|___|___|___| কৃষি জমিঃ<br>কানিঃ______ গণ্ডাঃ______কড়াঃ______= শতাংশ|___|___|___|___|

2.8	উল্লেখিত দ্রব্যসামগ্রী/ সম্পদগুলি কি আপনার খানায় আছে? (একাধিক উত্তর হতে পারে)	**No.**	পণ্য/ দব্যসামগ্রী	হ্যাঁ	না
		1	গরু/ ছাগল	1	2
		2	মাছ ধরার জাল	1	2
		3	হাঁস / মুরগী	1	2
		4	মুদি দোকান	1	2
		5	রিক্সা / ভ্যান / অটো রিক্সা	1	2
		6	কৃষি যন্ত্রপাতি	1	2
		7	নৌকা	1	2
		8	খাট / চৌকি	1	2
		9	কাথা / কম্বল	1	2
		10	তোষক	1	2
		11	হারিকেন	1	2
		12	চেয়ার / টেবিল	1	2
		13	ডাইনিং টেবিল	1	2
		14	আলমারি / শোকেস	1	2
		15	শোফা সেট	1	2
		16	টেলিভিশন	1	2
		17	রেডিও / টেপ রেকর্ডার	1	2
		18	ঘড়ি / দেওয়াল ঘড়ি	1	2
		19	টেলিফোন / মোবাইল ফোন	1	2
		20	বাইসাইকেল	1	2
		21	মোটর সাইকেল	1	2
		22	ফ্রিজ	1	2
		23	ফ্যান	1	2
		24	সেলাই মেশিন	1	2
		25	অন্যান্য (উল্লেখ করুন) ________________________________	1	2
2.9	আপনার কোন ধরনের পায়খানা ব্যবহার করেন?	নং	পায়খানার ধরন	হ্যাঁ	না
		1	সেনিটারী	1	2
		2	পীট পায়খানা	1	2
		3	পাকা পায়খানা	1	2
		4	কাঁচা পায়খানা	1	2
		5	অন্যান্য (উল্লেখ করুন)______________________	1	2
2.10	আপনাদের খাবার পানির মূল উৎস কি?	**নং**	উৎসের ধরন	হ্যাঁ	না
		1	টিউবওয়েল	1	2
		2	পুকুর/নদী/ডোবা / নালা	1	2
		3	বিশুদ্ধকৃত/ফিল্টার পানি	1	2
		4	বৃষ্টির পানি	1	2
		5	পাইপ/ ট্যাঁপ/ সাপ্লাই পানি	1	2
		6	অন্যান্য (উল্লেখ করুন)_____________________	1	2

**Section 3:** খানার সদস্যদের তথ্য											
কঃ 1	কঃ 2	কঃ 3	কঃ 4	কঃ 5	কঃ 6	কঃ 7	কঃ 8	কঃ 9	কঃ 10	কঃ 11	কঃ 12
(সদস্য কোড)	নাম<br>(সংক্ষিপ্ত নাম লিখুন)	খানা প্রধানের সাথে সম্পর্ক<br>1 খানা প্রধান নিজে<br>2 স্বামী/স্ত্রী<br>3 ভাই/বোন<br>4 ছেলে/মেয়ে/জামাতা/পুত্রবধু<br>5 মাতা/পিতা/শ্বশুর/শাশুড়ি<br>6 দাদা/দাদী<br>7 নাতি/নাতনী<br>8 চাচী/মামি/খালা/ফুফু<br>9 আত্মীয়-স্বজন নয় এমন<br>10 অন্যান্য(উল্লেখ করুন)	লিঙ্গ<br>1 পুরুষ<br>2 মহিলা	বয়স<br>(পূর্ণবছর)	বৈবাহিক অবস্থা<br>1 বিবাহিত<br>2 অবিবাহিত<br>3 বিপত্নীক/বিধবা<br>4 তালাক প্রাপ্ত<br>5 পৃথকীকৃত (separated)	শিক্ষাগত যোগ্যতা*	প্রধান পেশা<br>1 শ্রমিক<br>2 রিক্সা/ভ্যান চালক<br>3 অটো/সিএনজি চালক<br>4 রেস্টুরেন্ট কর্মী<br>5 দোকান কর্মচারী<br>6 গার্মেন্টস কর্মী<br>7 দর্জি<br>8 নাপিত<br>9 মুদী/চায়ের দোকান<br>10 ভ্রাম্যমাণ ব্যাবসা<br>11 চাকুরী<br>12 খামার(কৃষি, মৎস, দুগ্ধ ও মুরগী)<br>13 বর্গাচাষ<br>14 জেলে/মাঝি/মৎসচাষী<br>15 ক্ষুদ্র কুঠির শিল্প<br>16 দিনমজুর<br>17 মিস্ত্রী/ইলেক্ট্রিশিয়ান<br>18 গৃহকর্মী<br>19 গৃহিণী<br>20 বেকার<br>21 প্রতিবন্ধি<br>22 ছাত্রছাত্রী<br>23 শিশু/বয়স্ক<br>24 অন্যান্য (উল্লেখ করুন)	আপনি গত এক মাসে কতদিন কাজ করেছিলেন?	মাসিক আয় (টাকায়)	ল্যাম্পের সেবাভুক্তি<br>1 সেবাভুক্ত<br>2 সেবাভুক্ত নয়	কোন সদস্য কি কোন এনজিও, সমিতির অথবা বীমার সদস্য/সুবিধাভোগী?<br>1. হ্যাঁ<br>2. না<br>1 গ্রামীন ব্যাংক<br>2 ব্রাক<br>3 আশা<br>4 সেইফ<br>5 টিএমএমএস<br>6 বীমা<br>7 অন্যান্য(উল্লেখ করুন)<br>সমিতি বা এনজিও কোড

*কোডঃ নিরক্ষর/প্রাতিষ্ঠানিক শিক্ষা নাই (১), প্রাথমিক/পিএসসি অসমাপ্ত (২), প্রাথমিক/পিএসসি সমাপ্ত(৩), অষ্টম শ্রেণী/জিএসসি অসমাপ্ত (৪), অষ্টম শ্রেণী/জিএসসি সমাপ্ত (৫), মাধ্যমিক/দাখিল অসমাপ্ত (৬), মাধ্যমিক/দাখিল সমাপ্ত (৭), উচ্চ মাধ্যমিক/আলিম অসমাপ্ত (৮), উচ্চ মাধ্যমিক/আলিম সমাপ্ত (৯), স্নাতক/সম্মান/ফাজেল অসমাপ্ত (১০), স্নাতক/সম্মান/ফাজেল সমাপ্ত (১১), মাষ্টার্স ডিগ্রী/কামিল অসমাপ্ত (১২), মাষ্টার্স ডিগ্রী/কামিল সমাপ্ত (১৩)

Section 4: খানার ব্যয় সংক্রান্ত তথ্য				
**Part A: খাদ্য (সাপ্তাহিক ব্যয়)**				
কঃ 1	কঃ 2	কঃ 3	কঃ 4	কঃ 5
ক্রঃ নং	খাদ্যের ধরন	ভোগের/খাওয়ার পরিমান	ব্যয় (বাজার দর)টাকা অনুযায়ী	উৎস<br>(1=ক্রয়কৃত,<br>2=কাজের বিনিময়ে,<br>3=নিজে উৎপাদিত, 4=উপহার)
**4.1** গত এক সপ্তাহে খাদ্য শস্য				
1	চাল (কেজি)			
2	আটা(কেজি)			
3	ময়দা (কেজি)			
4	চিড়া/মুড়ি (কেজি)			
**4.2** গত এক সপ্তাহে ডাল জাতীয় শস্য				
5	মশুর ডাল (কেজি)			
6	ছোলার ডাল (কেজি)			
7	মাস কালাই (কেজি)			
8	খেসারী (কেজি)			
9	মুগ (কেজি)			
10	বুট (কেজি)			
11	এ্যাংকর (কেজি)			
**4.3** গত এক সপ্তাহে ভোজ্য তৈল				
12	সয়াবিন (কেজি)			
13	সরিষা (কেজি)			
14	ডালডা (কেজি)			
15	ঘি (কেজি)			
16	পাম অয়েল (কেজি)			
17	অন্যান্য (কেজি)___________________			
**4.4** এক সপ্তাহে শাক/ সবজি				
18	লাল শাক (কেজি)			
19	পুঁই শাক (কেজি)			
20	পালং শাক (কেজি)			
21	কলমি শাক (কেজি)			
22	অন্যান্য শাক ____________ (কেজি)			
23	অন্যান্য শাক ____________ (কেজি)			
24	অন্যান্য শাক ____________ (কেজি)			
25	আলু (কেজি)			
26	পটল (কেজি)			
27	করলা			
28	ফুলকপি			
29	বাধাকপি			
30	বেগুন (কেজি)			
31	কচু			
32	লাউ			
33	টমেটো (কেজি)			
34	কাঁচা পেপে (কেজি)			
35	কাঁচা কলা (কেজি)			
36	কাঁচা মরিচ (কেজি)			
37	পেয়াজ (কেজি)			
38	রসুন (কেজি)			
39	আদা (কেজি)			
40	অন্যান্য সবজি____________ (কেজি)			
41	অন্যান্য সবজি____________ (কেজি)			
42	অন্যান্য সবজি____________ (কেজি)			
**Part B: খাদ্য (মাসিক ব্যয়)**				
**4.5** গত এক মাসে মসলা				
1	শুকনা মরিচ (গ্রাম)			
2	হলুদ (গ্রাম)			
3	জিরা (গ্রাম)			
4	দারুচিনি (গ্রাম)			
5	এলাচি (গ্রাম)			
6	অন্যান্য মসলা_____________(গ্রাম)			
7	অন্যান্য মসলা_____________(গ্রাম)			
8	অন্যান্য মসলা_____________(গ্রাম)			
**4.6** গত এক মাসে মাছ, মাংস, ডিম ও দুধ				
9	বড় মাছ (কেজি)			
10	ছোট মাছ (কেজি)			
11	শুটকি মাছ (কেজি)			
12	গরুর মাংস (কেজি)			
13	মহিষের মাংস (কেজি)			
14	দেশী মুরগীর মাংস (কেজি)			

ক্রঃ নং	খাদ্যের ধরন (একক)	ভোগের/খাওয়ার পরিমান	ব্যয় (বাজার দর)টাকা অনুযায়ী	উৎস (1=ক্রয়কৃত, 2=কাজের বিনিময়ে, 3=নিজে উৎপাদিত, 4=উপহার)
15	ফার্ম মুরগীর মাংস (কেজি)			
16	হাঁসের মাংস (কেজি)			
17	খাসীর মাংস (কেজি)			
18	ডিম (সংখ্যা)			
19	দুধ (কেজি)			
20	অন্যান্য আমিষ জাতীয় খাবার________(কেজি)			
21	অন্যান্য আমিষ জাতীয় খাবার________(কেজি)			
22	অন্যান্য আমিষ জাতীয় খাবার________(কেজি)			
**4.7** গত এক মাসে ফল				
23	কলা (সংখ্যা)			
24	বড়ই(কেজি)			
25	জলপাই (কেজি)			
26	পাকা পেপে (কেজি)			
27	আম (কেজি)			
28	কাঁঠাল (সংখ্যা)			
29	জাম (কেজি)			
30	আপেল (কেজি)			
31	কমলা (কেজি)			
32	আংগুর (কেজি)			
33	অন্যান্য ফল ____________ (কেজি)			
34	অন্যান্য ফল ____________ (কেজি)			
35	অন্যান্য ফল ____________ (কেজি)			
**4.8** গত এক মাসে অন্যান্য খাবার				
36	চিনি (কেজি)			
37	গুর (কেজি)			
38	গুঁড়ো দুধ (কেজি)			
39	লবন (কেজি)			
40	নুডুলস (কেজি)			
41	সুজি/সেমাই (কেজি)			
42	কোমল পানীয় (লিটার)			
43	মিষ্টি জাতীয় খাদ্য (কেজি)			
44	শিশু খাদ্য______________ (কেজি)			
45	চা পাতা (কেজি)			
46	বিস্কুট (কেজি)			
47	চানাচুর (কেজি)			
48	অন্যান্য ____________ (কেজি)			
49	অন্যান্য ____________ (কেজি)			
50	অন্যান্য ____________ (কেজি)			
**Part C: তামাকজাত দ্রব্য (সপ্তাহিক ব্যয়)**				
**4.9** গত এক সপ্তাহে তামাকজাত দ্রব্য ভোগের পরিমান				
1	পান (সংখ্যা)			
2	সুপারী (সংখ্যা)			
3	জর্দা (কৌটা)			
4	তামাক (সংখ্যা)			
5	গুল (কৌটা)			
6	বিড়ি (প্যাকেট)			
7	সিগারেট (প্যাকেট)			
8	অন্যান্য ____________ (কেজি)			
9	অন্যান্য ____________ (কেজি)			
10	অন্যান্য ____________ (কেজি)			

Part D: খাদ্য বাদে (মাসিক ব্যয়)

কঃ 1	কঃ 2	কঃ 3
ক্র: নং	খাত	খরচের পরিমান (মাসিক)
**4.10** গত এক মাসে খাদ্য বাদে ব্যয়		
1	ভাড়া	
2	বিদ্যুত বিল/ সৌর বিদ্যুৎ	
3	গ্যাস বিল	
4	জ্বালানি কাঠ	
5	জ্বালানি তৈল (কেরোসিন, পেট্রোল..)	
6	পরিবহন	
7	স্বাস্থ্য	
8	শিক্ষা (স্কুলের বেতন, কোচিং, প্রাইভেট টিচার ইত্যাদি)	

কঃ 1	কঃ 2	কঃ 3
**ক্র: নং**	**খাত**	**খরচের পরিমান (মাসিক)**
9	খাতা/কলম/বই/ অন্যান্য শিক্ষাউপকরন	
10	যোগাযোগ ব্যয় (মোবাইল, টেলিফোন ইত্যাদি)	
11	ডিশ বিল/ইন্টারনেট ব্যয়	
12	কসমেটিক ব্যয় (ক্রিম, পাউডার, মেকাপ ইত্যাদি)	
13	চুলে ব্যবহারের তেল (নাড়িকেল তেল)	
14	পরিষ্কার ও পরিচ্ছন্নতা ব্যয় (সাবান, ডিটারজেন্ট, শ্যম্পু ইত্যাদি)	
15	লন্ড্রি	
16	অর্থের বহির্গমন (যেকোনো প্রকার)	
17	সমিতির কিস্তি/ক্ষুদ্রঋনের কিস্তি	
18	সেলুন/পার্লার	
19	অন্যান্য (নির্দিষ্ট করে বলুন)	

Part C: খাদ্য বাদে (বার্ষিক ব্যয়)

**4.11** গত এক বছরে খাদ্য বাদে ব্যয়		
1	পোশাক	
2	জুতা	
3	লেপ/তোষক/বালিশ/চাদর/বিছানা	
4	বিনোদন / ধর্মীয় অনুষ্ঠান	
5	গৃহ নির্মাণ/গৃহ সংস্কার	
6	আসবাবপত্র	
7	ইন্সুরেন্স প্রিমিয়াম	
8	গৃহপরিচালক / গৃহপরিচারিকার বেতন	
9	উপহার প্রদান	
10	দান/ফিতরা	
11	ভ্রমন	
12	অন্যান্য(নির্দিষ্ট করে বলুন)	

Section 5: ল্যাম্পের সেবা গ্রহন সংক্রান্ত তথ্য

**5.1**	বিগত তিনমাসে ল্যাম্পের সেবা গ্রহনের পরিমান				
	**সেবা**	**সেবা গ্রহনকারীর কোড**	**সেবার পরিমান**	**সেবা গ্রহনে মোট ব্যয়**	**একই সেবা অন্যত্র গ্রহনে ব্যয়/বাজার মুল্য**
1	এমবিবিএস ডক্তারের পরামর্শ				
2	স্যাটেলাইট ক্লিনিকে সেবা				
3	হেল্থ ক্যাম্পে সেবা				
4	স্পেসিয়ালিস্ট ডক্তারের পরামর্শ				
5	ডায়াগনস্টিক টেস্ট				
6	হসপাতালে চিকিৎসা গ্রহনে ভর্তুকী				
7	ঔষধে ভর্তুকী				
8	অন্যন্য স্বাস্থ্যসেবা______				
9	কম্পিউটার প্রশিক্ষন				
		**পন্য**	**পরিমান**	**সেবা গ্রহনে মোট ব্যয়**	**একই পন্য অন্যত্র মুল্য/বাজার মুল্য**
10.	ল্যাম্প স্টোর সেবা	চাল			
		ডাল			
		ময়দা			
		চিনি			
		সয়াবিন			

**5.2**	ল্যাম্পের সেবা গ্রহন না করে থাকলে তার কারন		
**1. সেবা**	**☐ সাস্থ্যসেবা গ্রহন করেননি**	**☐ পরিবারের কোন সদস্য কম্পিউটার প্রশিক্ষন গ্রহন করেননি**	**☐ ল্যাম্প স্টোর থেকে পন্য**
2. গ্রহন না করার কারন	1. পরিবারের কোনো সদস্য অসুস্থ ছিলনা	1. পরিবারের কোনো প্রশিক্ষেনের যোগ্য শিশু কিশোর নেই	1. আপনার এলাকায় ল্যাম্প স্টোর নেই
	2. অসুস্থ ছিল কিন্তু জটিল কোনো সমস্যা ছিল না	2. আপনার পরিবারের কোনো সদস্য আগ্রহী নয়	2. ল্যাম্প স্টোরে বাকীতে পন্য দেয়া হয়না
	3. ল্যম্পে চিকিৎসার খরচ অত্যধিক	3. প্রশিক্ষনের খরচ অত্যধিক	3. অন্য স্টোরে বাকীতে পন্য কিনেন
	4. ল্যম্পে চিকিৎসা গ্রহনের পর্যাপ্ত অর্থ ছিলনা	4. ল্যম্পে প্রশিক্ষেন গ্রহনের পর্যাপ্ত অর্থ ছিলনা	4. পরিচিত স্টোর হতে পন্য কিনেন
	5. ল্যাম্প স্বাস্থ্যকেন্দ্রের দূরত্ব খুব বেশি	5. প্রশিক্ষন সেবা সম্পর্কে অবহিত ছিলেননা	5. ল্যাম্প স্টোরের দূরত্ব খুব বেশি
	6. ল্যাম্প স্বাস্থ্যকেন্দ্রে নেয়ার জন্য কে ছিল না	6. প্রশিক্ষন পরবর্তী উপযুক্ত সময়ে নিবেন	6. ল্যাম্প স্টোরে সব পন্য নেই
	7. ল্যাম্প স্বাস্থ্যকেন্দ্রের অবস্থান জানেননা	7. ল্যাম্প কেন্দ্রের দূরত্ব খুব বেশি	7. ল্যাম্প স্টোরের অবস্থান জানেননা
	8. ল্যাম্প স্বাস্থ্যসেবার মান ভাল না	8. ল্যাম্প প্রশিক্ষনের মান ভাল না	8. ল্যাম্প স্টোরের পন্যের মান ভালো না
	9. অন্যান্য (উল্লেখ করুন____________________)	9. অন্যান্য (উল্লেখ করুন____________________)	9. অন্যান্য (উল্লেখ করুন____________________)

Section 6: খানার সদস্যদের বিগত ৩মাসে অসুস্থতা সম্পর্কিত তথ্য (একাধিক অসুস্থ্যতার তথ্য সদস্য কোডসহ ভিন্ন ভিন্ন সারিতে লিখুন)

গত তিন মাসে আপনি বা আপনার খানার কোন সদস্য কি কোন রোগের লক্ষণ/ গর্ভসংক্রান্ত সমস্যা/ সন্তান প্রসব হয়েছিল কি? □ হ্যাঁ (1), □ না (2) (হ্যাঁ হলে নিম্নোক্ত টেবিল টি পূরণ করুন)

কঃ 1	কঃ 2	কঃ 3	কঃ 4	কঃ 5	কঃ 6		কঃ 7	কঃ 8	কঃ 9										কঃ 10	কঃ 11					
(সদস্য কোড)	ঐ সদস্যের রোগের কোড লিখুন *	আপনি ঐ অসুখে কতদিন ভুগেছিলেন?	আপনি কি ঐ অসুখের জন্য কোন স্বাস্থ্যসেবা নিয়েছিলেন? কোডঃ ১= হ্যাঁ, ২= না (উত্তর যদি না হয় তবে পরবর্তী সেকশনে যান)	যদি চিকিৎসা না নিয়ে থাকেন, কি কারনে নেননি?<br>১.জটিল কোনো সমস্যা ছিল না<br>২.চিকিৎসার খরচ অত্যধিক<br>৩.পর্যাপ্ত অর্থ ছিলনা<br>৪.স্বাস্থ্যকেন্দ্রের দূরত্ব খুব বেশি<br>৫.স্বাস্থ্যকেন্দ্রে নেয়ার জন্য কেউ ছিল না<br>৬.স্বাস্থ্যকেন্দ্রের অবস্থান জানেননা<br>৭.স্বাস্থ্যসেবার মান ভাল না<br>৮.অন্যান্য (উল্লেখ করুন....)	আপনি কার কাছ থেকে চিকিৎসা নিয়েছিলেন?<br>1. পল্লী চিকিৎসক<br>2. ঔষধের দোকান<br>3. অন্যান্য এমবিবিএস ডাক্তার<br>4.কমিউনিটি ক্লিনিক<br>5. এনজিও ক্লিনিক<br>6. বেসরকারী ক্লিনিক<br>7.উপজেলা হেল্থ কমপ্লেক্স<br>8.জেলা মেডিকাল কলেজ/হাসপাতাল<br>9. ল্যাম্প সেন্টার<br>10. ল্যাম্প হেল্থ /স্যাটেলাইট ক্যাম্প<br>11.নিজে নিজে পরিবারের সদস্য দ্বারা চিকিৎসা<br>12.কবিরাজ/হোমিওপ্যাথ/<br>13. অন্যান্য (উল্লেখ করুন)		আপনার কি ঐ সমস্যা বা অসুখের জন্য হাসপাতালে ভর্তি হতে হয়েছিলো? কোডঃ 1= হ্যাঁ 2= না	যদি হ্যাঁ হয় তবে, কতদিন হাস্পাতালে ভর্তি ছিলেন? (পুর্নদিবস)	চিকিৎসা বাবদ খরচ<br>(যে ব্যয়টি প্রযোজ্য হবে না, সেক্ষেত্রে ‘0’ লিখুন)										অসুস্থতা জনিত কারনে কতদিন কাজে অনুপস্থিত ছিলেন (পুর্নদিবস)	আপনি চিকিৎসার খরচ কিভাবে জোগাড় করেছিলেন?<br>1. নিয়মিত আয়<br>2. পরিবারের সঞ্চয়<br>3. স্বাস্থ্যবীমা<br>4. স্বাস্থ্য ব্যাতীত অন্য কোনো বীমা<br>5. নিজস্ব জিনিসপত্র বিক্রি<br>6. গাছ/কৃষিপণ্য/গৃহপালিত প্রাণী বিক্রি<br>7. স্থায়ী সম্পত্তি বিক্রি<br>8. জমি বা সম্পত্তি বন্ধক<br>9. মহাজনের কাছ থেকে টাকা ধার<br>10. বন্ধু বা আত্মীয় স্বজনদের কাছ থেকে সাহায্য<br>11. আত্মীয়/বন্ধু/সহকর্মীদের কাছ থেকে ধার<br>12. কোন প্রতিষ্ঠান/সমিতি থেকে ঋণ গ্রহণ<br>13.অন্যান্য (নির্দিষ্ট করুন)					
					ক্রমিক নং	চিকিৎসার উৎস			পরামর্শ ফি	ঔষধ	বিছানা ভাড়া	পরীক্ষা খরচ	যাতায়াত	বকশিশ	এটেন্ডেন্ট খরচ	প্যাকেজ খরচ	অন্যান্য	মোট খরচ		উৎস -1	টাকা	উৎস -2	টাকা	উৎস -3	টাকা
					1.																				
					2.																				
					3.																				
					1.																				
					2.																				
					3.																				
					1.																				
					2.																				
					3.																				
					1.																				
					2.																				
					3.																				
					1.																				
					2.																				
					3.																				

*1=সর্দি কাশি, 2=জ্বর,3=টাইফয়েড, 4=ডায়রিয়া/পাতলা পায়খানা/আমাশয়, 5=বমি, 6=হাঁপানি/শ্বাসকষ্ট, 7=ছোট কাঁটাছেঁড়া,8=হাড়ভাঙ্গা 9=জন্ডিস, 10=দুর্বলতা, 11=কৃমি, 12=ব্যাথা, 13=চর্মরোগ, 14=খিঁচুনি, 15=বুকজ্বালা, 16=উচ্চ রক্তচাপ, 17=ডায়াবেটিস, 18=যক্ষ্মা, 19=কুষ্ঠরোগ, 20=পক্ষাঘাত/প্যারালাইসিস, 21=বাত, 22=বাতজ্বর 23= ক্যান্সার, 24= সন্তান প্রসব, 25= গর্ভসংক্রান্ত সমস্যা 26= মানসিক সমস্যা, 27= অন্যান্য (নির্দিষ্ট করুন)

**Section 7: খানার উপর ল্যাম্প সদস্যের অসুস্থতার প্রভাব**<br>(যদি ল্যাম্প সদস্য অসুস্থতায় না ভোগেন ধন্যবাদ জানিয়ে সাক্ষাৎকার শেষ করুন)			
**Part 7A: অসুস্থতা থাকাকালে উপস্থিতি**			
1	ল্যাম্প সদস্য বিগত 3 মাসের মধ্যে অসুস্থ থাকাকালে কতদিন কাজে উপস্থিত ছিলেন? (প্রযোজ্য না হলে “NA” লিখুন)		
**Part 7B: কাজ থেকে অসুস্থতার কারণে ড্রপ-আউট**			
2	ল্যাম্প সদস্য গত 3 মাসের মধ্যে অসুস্থতার কারণে স্থায়ীভাবে/ সাময়িক ভাবে কোনো কাজ ছেড়েছেন কি?	☐ হ্যাঁ (1)<br>☐ না (2)<br>☐ প্রযোজ্য নয় (99)	
**Part 7C: খাদ্য নিরাপত্তার উপর অসুস্থতার প্রভাব**			
3	গত 3 মাসের মধ্যে ল্যাম্প সদস্যের অসুস্থতার খরচের জন্য আপনার পরিবারের খাদ্য গ্রহণ ও অন্যান্য খরচ কমাতে হয়েছিল কি?	☐ হ্যাঁ (1)<br>☐ না (2)<br>☐ প্রযোজ্য নয় (99)	
**Part 7 D: অসুস্থতার কারণে শিশুর স্কুল থেকে ড্রপ-আউট**			
4	গত 3 মাসের মধ্যে ল্যাম্প সদস্যের অসুস্থতার কারণে আপনার পরিবারের কোনো শিশু সদস্যদের স্থায়ীভাবে স্কুল ছাড়তে হয়েছিল কি?	☐ হ্যাঁ (1)<br>☐ না (2)<br>☐ প্রযোজ্য নয় (99)	সদস্য কোডঃ
**Part 7 E: অসুস্থতার কারণে শিশু শ্রম**			
5	গত 3 মাসের মধ্যে ল্যাম্প সদস্যের অসুস্থতার কারণে আপনার পরিবারের কোনো শিশু সদস্যদের কাজে যোগ দিতে হয়েছিল কি?	☐ হ্যাঁ (1)<br>☐ না (2)<br>☐ প্রযোজ্য নয় (99)	সদস্য কোডঃ
**Section 8: ল্যাম্প সদস্যের স্বাস্থ্যসেবায় সন্তুষ্টি**<br>(সাক্ষাৎপ্রদানকারী ল্যাম্প সদস্য হলে / ল্যাম্প সদস্যের স্বাস্থ্যসেবা গ্রহনের সময় সংগে থাকলে এই প্রশ্নগুলি করুন)			
1	চিকিৎসাকেন্দ্রের ডাক্তারের ব্যবহার সম্পর্কে আপনার মতামত কি?	1. খুব খারাপ<br>2. খারাপ<br>3. মোটামুটি<br>4. ভালো<br>5. খুব ভালো	
2	ডাক্তারের নির্দেশিত ঔষধগুলো কখন কিভাবে খেতে হবে এবং অন্যান্য উপদেশগুলো কিভাবে পালন করতে হবে সেটা কি আপনাকে বুঝিয়ে দেয়া হয়েছিল?	1. একদমই ব্যাখ্যা করা হয়নি<br>2. আংশিক ব্যাখ্যা করা হয়েছে<br>3. মোটামুটি ব্যাখ্যা করা হয়েছে<br>4. ব্যাখ্যা করা হয়েছে<br>5. সম্পূর্ণভাবে ব্যাখ্যা করা হয়েছে	
3	আপনাকে যে টেস্টগুলো করতে দেয়া হয়েছে সেগুলো কেন করা দরকার, সেটা কি আপনার কাছে ব্যাখ্যা করে বলা হয়েছিল?	1. একদমই ব্যাখ্যা করা হয়নি<br>2. আংশিক ব্যাখ্যা করা হয়েছে<br>3. মোটামুটি ব্যাখ্যা করা হয়েছে<br>4. ব্যাখ্যা করা হয়েছে<br>5. সম্পূর্ণভাবে ব্যাখ্যা করা হয়েছে	
4	চিকিৎসাকেন্দ্রের ডাক্তারবাদে অন্যান্য স্টাফের ব্যবহার সম্পর্কে আপনার মতামত কি?	1. খুব খারাপ<br>2. খারাপ<br>3. মোটামুটি<br>4. ভালো<br>5. খুব ভালো	
5	অভ্যর্থনা কক্ষ, টয়লেট এবং অন্যান্য উপকরনসমূহ পরিস্কার পরিচ্ছন্ন ছিল কি?	1. খুব অপরিচ্ছন্ন ছিল<br>2. অপরিচ্ছন্ন ছিল<br>3. মোটামুটি পরিচ্ছন্ন ছিল<br>4. পরিচ্ছন্ন ছিল<br>5. খুব পরিচ্ছন্ন ছিল	
6	চিকিৎসাকেন্দ্রের সার্বিক সেবা প্রদান সম্পর্কে আপনার অনুভূতি কি?	1. খুব খারাপ<br>2. খারাপ<br>3. মোটামুটি<br>4. ভালো<br>5. খুব ভালো	

আপনার মূল্যবান সময় দেয়ার জন্য আপনাকে ধন্যবাদ
